# Group X secreted phospholipase A_2_ induces lipid droplet formation and prolongs breast cancer cell survival

**DOI:** 10.1186/1476-4598-12-111

**Published:** 2013-09-27

**Authors:** Anja Pucer, Vesna Brglez, Christine Payré, Jože Pungerčar, Gérard Lambeau, Toni Petan

**Affiliations:** 1 Department of Molecular and Biomedical Sciences, Jožef Stefan Institute, Ljubljana, Slovenia; 2 Institut de Pharmacologie Moléculaire et Cellulaire, CNRS et Université de Nice Sophia Antipolis, UMR 6097, Sophia Antipolis Valbonne, France

**Keywords:** Secreted phospholipase A_2_, Breast cancer, Cell survival, Apoptosis, Lipid droplets, Fatty acid oxidation, Lipid signaling, Etomoxir, Carnitine palmitoyltransferase 1, AMP-activated protein kinase

## Abstract

**Background:**

Alterations in lipid metabolism are inherent to the metabolic transformations that support tumorigenesis. The relationship between the synthesis, storage and use of lipids and their importance in cancer is poorly understood. The human group X secreted phospholipase A_2_ (hGX sPLA_2_) releases fatty acids (FAs) from cell membranes and lipoproteins, but its involvement in the regulation of cellular FA metabolism and cancer is not known.

**Results:**

Here we demonstrate that hGX sPLA_2_ induces lipid droplet (LD) formation in invasive breast cancer cells, stimulates their proliferation and prevents their death on serum deprivation. The effects of hGX sPLA_2_ are shown to be dependent on its enzymatic activity, are mimicked by oleic acid and include activation of protein kinase B/Akt, a cell survival signaling kinase. The hGX sPLA_2_-stimulated LD biogenesis is accompanied by AMP-activated protein kinase (AMPK) activation, up-regulation of FA oxidation enzymes and the LD-coating protein perilipin 2, and suppression of lipogenic gene expression. Prolonged activation of AMPK inhibited hGX sPLA_2_-induced LD formation, while etomoxir, an inhibitor of FA oxidation, abrogated both LD formation and cell survival. The hGX sPLA_2_-induced changes in lipid metabolism provide a minimal immediate proliferative advantage during growth under optimal conditions, but they confer to the breast cancer cells a sustained ability to resist apoptosis during nutrient and growth factor limitation.

**Conclusion:**

Our results identify hGX sPLA_2_ as a novel modulator of lipid metabolism that promotes breast cancer cell growth and survival by stimulating LD formation and FA oxidation.

## Background

Tumor cells display progressive, oncogene-driven alterations in the metabolic pathways that supply energy and biosynthetic intermediates to enable their survival, growth and proliferation [1]. At the core of this metabolic reprogramming is a shift towards macromolecular biosynthesis, based largely on the use of mitochondrial metabolites as anabolic precursors, and supported by changes in lipid synthesis, degradation and signaling [2-4]. The attainment of a lipogenic phenotype, characterized by the increased dependence of cancer cells on *de novo* fatty acid (FA) synthesis, is typical of many cancer cells [2]. The transformed properties of tumor cells can also depend on lipolytic remodeling [3,5] and FA oxidation [6-10]. The biochemical mechanisms governing the transformations of lipid metabolism in cancer cells, in particular the relationships between lipid synthesis, storage and use, and their importance in the neoplastic process are still largely unknown. Identifying the factors responsible for the modulation of lipid metabolism and signaling in cancer is important for understanding the disease and for devising more rational preventive and therapeutic approaches.

Secreted phospholipases A_2_ (sPLA_2_s) are lipolytic enzymes that act on membrane glycerophospholipids to liberate free FAs (FFAs) and lysophospholipids by catalyzing the hydrolysis of their *sn*-2 ester bond [11]. These low-molecular mass, disulfide-rich and Ca^2+^-dependent enzymes are secreted from a variety of cells and act in autocrine or paracrine manners on cell membranes and other extracellular phospholipids, including lipoprotein particles, surfactant and dietary lipids, microbial membranes and microvesicles [12]. The nine active sPLA_2_ enzymes known in humans display different tissue expression patterns and specific enzymatic preferences for binding to different types of phospholipid membranes, suggesting distinct biological roles for each sPLA_2_[13,14]. The multitude of cellular effects of the released FAs and lysophospholipids, and of their numerous bioactive metabolites, further explain their involvement in a variety of physiological processes and diseases, including lipid digestion and remodeling, acute and chronic inflammatory diseases, cardiovascular diseases, reproduction and host defense against infections [12]. Recent studies have implicated various sPLA_2_s in cancer and metabolic disorders [15].

Aberrant expression of various sPLA_2_s in cancer cells has been associated with the pathology of colorectal, breast, gastric and prostate cancers [16,17]. The most studied group IIA sPLA_2_ has been proposed to have a pro-tumorigenic role in prostate [18] and esophageal cancer [19], but an anti-tumorigenic role in gastric cancer [20]. Its role in colorectal cancer is still controversial [12,16,21,22]. The involvement of sPLA_2_s in cancer and other diseases has been investigated in relation to their ability to release arachidonic acid (AA) from cell membranes and stimulate, either directly or in coordination with the cytosolic group IVA PLA_2_ (cPLA_2_α), the production of eicosanoids, including the mitogenic prostaglandin E2 (PGE2) [12,14,23]. Several studies have suggested a tumor-promoting role for the group III and X sPLA_2_s in colorectal cancer, based on their ability to stimulate PGE2 synthesis and cell proliferation [24,25]. However, the human group X (hGX) sPLA_2_ stimulates colon cancer cell proliferation by a mechanism that is dependent on the released FFAs and lysophospholipids, but not on its potent stimulation of PGE2 synthesis [26]. The underlying mechanisms of the action of hGX sPLA_2_ and other sPLA_2_ enzymes in different cancers are not known and confirmation of their functional contribution to tumorigenesis awaits further studies.

The group X sPLA_2_ displays the greatest potency among mammalian sPLA_2_s in hydrolyzing the phosphatidylcholine (PC)-rich extracellular leaflet of mammalian plasma membranes and of lipoprotein particles [12,13]. Besides AA, the enzyme also releases numerous other monounsaturated and polyunsaturated FFAs, which could influence lipid metabolism and tumorigenesis in a variety of ways [3,7,12,27,28]. FFAs can be remodeled into membrane phospholipids, catabolized through mitochondrial FA oxidation or esterified into triacylglycerols (TAGs) and stored in lipid droplets (LDs) [29-31]. Several enzymes regulating FFA availability through synthesis, such as fatty acid synthase (FAS) and acetyl-CoA carboxylase (ACC) [2], and through lipolysis [3,5] have been clearly associated with cancer. In addition, there is increasing evidence for an important role for mitochondrial FA oxidation in tumorigenesis [6,8-10]. Interestingly, several recent reports have revealed that group X sPLA_2_ affects lipid metabolism in various physiological and pathophysiological settings, including steroid hormone synthesis in adrenal glands [32], lipid digestion in the gut and diet-induced obesity [33]. Its recently proposed role in adipogenesis in mice has been associated with down-regulation of the expression of several genes important for lipid synthesis and adipogenesis, including sterol regulatory element-binding protein-1 (SREBP-1) and FAS [34]. Additionally, the group X sPLA_2_ hydrolyzes serum low-density lipoprotein (LDL) and stimulates lipid accumulation and foam cell formation from macrophages [12]. The possible associations between sPLA_2_s and basic lipid metabolism, such as fatty acid oxidation and synthesis, TAG synthesis and lipolysis, in the context of cell fate and tumorigenesis have, however, not been explored.

Altered lipid metabolism, including lipogenesis, β-oxidation and phospholipid remodeling, contributes to the transformed phenotype of breast cancer [2,4,35]. The involvement of sPLA_2_s in breast cancer has not been studied, and there are only a few reports correlating the increased expression of group IIA sPLA_2_ with advanced cancer and decreased patient survival [17,36]. The aim of this study was to determine whether hGX sPLA_2_ affects breast cancer cell growth and survival, and to delineate the underlying mechanism of action. We show for the first time that hGX sPLA_2_ induces LD formation in the highly tumorigenic MDA-MB-231 breast cancer cells in an enzyme activity-dependent manner, thereby stimulating cell proliferation and significantly prolonging cell survival under serum deprivation-induced stress. Our results suggest that FFAs, in particular oleic acid (OA), released from membrane phospholipids by the action of hGX sPLA_2_, are in large part responsible for LD biogenesis and cell survival. We also demonstrate that the mechanism of hGX-induced cell survival and lipid accumulation is associated with alterations in the expression of key lipogenic and β-oxidation enzymes, and modulation of AMP-activated protein kinase (AMPK) and protein B/Akt kinase signaling pathways. The pro-tumorigenic effects induced by hGX sPLA_2_ were abolished by etomoxir, suggesting a critical role for β-oxidation in hGX-induced LD formation and cell survival in breast cancer cells.

## Results

### hGX sPLA_2_ stimulates proliferation and prolongs serum-free survival of MDA-MB-231 cells in an enzyme activity-dependent manner

**Figure 1:**
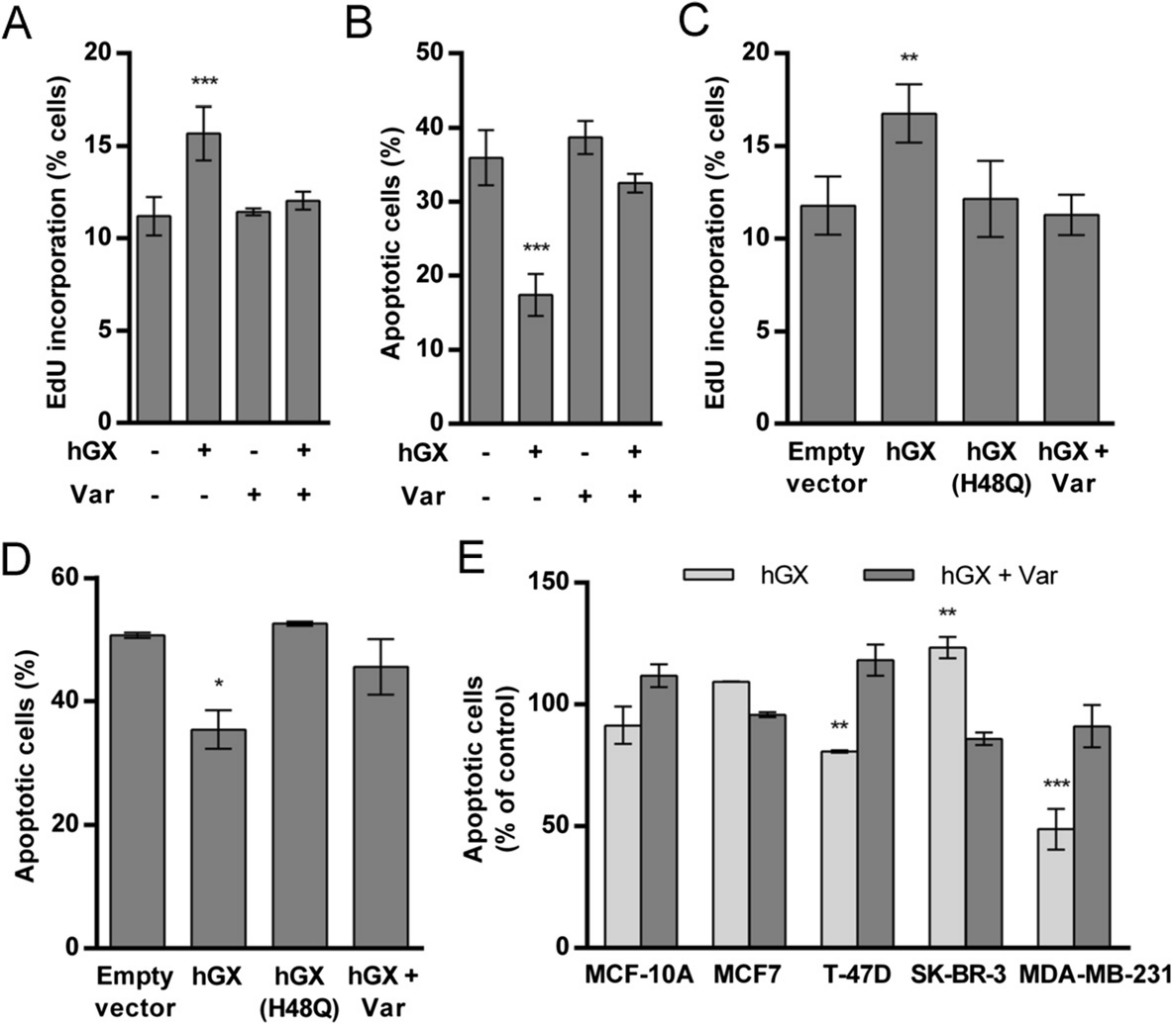
**hGX sPLA**_**2 **_**stimulates the proliferation and prevents serum withdrawal-induced cell death of breast cancer cells in an enzymatic activity-dependent manner.** After serum deprivation for 48 h **(A)** or 24 h **(B)** MDA-MB-231 cells were treated with recombinant hGX (10 nM) in serum-free medium containing 0.1% FAF BSA for 24 h **(A)** or 96 h **(B)** in the presence or absence of the sPLA_2_ inhibitor varespladib (Var) at a final concentration of 50 μM. MDA-MB-231 cells grown for 24 h in complete culture medium were transiently transfected with empty vector and plasmids encoding the wild-type hGX or catalytic-site mutant hGX(H48Q) **(C, D)**. The cells were then cultured in complete medium in the presence or absence of varespladib for an additional 48 h **(C)**. Alternatively, the cells were washed at 24 h post-transfection, and incubated in serum-free medium containing 0.05% FAF BSA for an additional 96 h **(D)**. Breast cancer cell lines were exposed to prolonged serum-deprivation without medium renewal, as described in Methods, and treated with 10 nM recombinant hGX in the presence or absence of 50 μM varespladib **(E)**. Cell proliferation **(A, C)** was determined using the EdU incorporation assay on fixed cells with additional 7-AAD staining for accurate quantification of cells in the S-phase of the cell cycle. Cell death **(B, D, E)** was analyzed using the TMRM/YO-PRO-1 assay and the percentage of TMRM negative and YO-PRO-1 positive (late apoptotic) cells within the population was used for final analyses. The resulting values are means ± SD of at least two experiments performed in duplicate. Results that are statistically significant over control samples are indicated (*, P < 0.05; **, P < 0.01; ***, P < 0.001; one-way ANOVA with Bonferroni adjustment).

In order to determine whether hGX sPLA_2_ affects the growth of breast cancer cells, we measured the proliferation rate of MDA-MB-231 cells treated with hGX sPLA_2_. Addition of recombinant hGX sPLA_2_ (10 nM) stimulated the proliferation of quiescent, serum-deprived MDA-MB-231 cells (Figure 1A). The effect was completely abolished by the sPLA_2_ inhibitor varespladib [37,38], suggesting a dependence on sPLA_2_ enzyme activity. Importantly, the enzyme also displayed a mitogenic effect at sub-nanomolar concentrations in proliferating MDA-MB-231 cells grown in the presence of 10% FBS (Additional file 1: Figure S1A). To confirm the dependence on enzymatic activity of hGX-induced stimulation of cell proliferation, we treated starved MDA-MB-231 cells with a range of concentrations of recombinant wild-type mouse group X (mGX) sPLA_2_ and with its active site mutant mGX H48Q that possesses less than 0.1% of the wild-type enzyme activity [26]. The use of the mouse ortholog of hGX sPLA_2_ is justified since both enzymes display very similar enzymatic characteristics on cell membranes and mitogenic activities on colon cancer cells [13,26]. The results (Additional file 1: Figure S1B) confirm the role of enzyme activity in the mitogenic effect of the group X sPLA_2_ enzyme, since mGX sPLA_2_ induced a similar increase in the rate of cell proliferation as the human enzyme, whereas its catalytically inactive H48Q mutant did not induce significant changes in MDA-MB-231 cell proliferation. The inability of the mutant to stimulate cell proliferation also excludes a potential mitogenic action of a low-level contaminating agent, such as lipopolysaccharide (LPS), which may be present in bacterially expressed recombinant sPLA_2_s.

Since the positive effect of hGX sPLA_2_ on MDA-MB-231 cell proliferation was more prominent when the cells were serum-starved, we questioned whether the apparent mitogenic effect of hGX sPLA_2_ could be the result of an increase in cell survival under conditions of serum and nutrient limitation. Indeed, when MDA-MB-231 cells were serum-starved for 24 h and then incubated with recombinant hGX sPLA_2_, in the absence of serum and without medium renewal for the next 96 h, there was a 2-fold reduction in the percentage of late apoptotic cells in treated cells relative to untreated controls (Figure 1B) and a corresponding 2-fold increase in the number of healthy adherent cells (Additional file 1: Figure S1C). This robust anti-apoptotic effect was completely prevented by inhibition of the enzyme with varespladib, suggesting that the ability of hGX sPLA_2_ to prevent MDA-MB-231 cell death during prolonged serum withdrawal is dependent on the products of its hydrolysis. Together, the above results using exogenously added sPLA_2_ show that hGX sPLA_2_ can act from the extracellular milieu to exert a pro-survival effect via its enzymatic activity.

Exogenously added and ectopically expressed sPLA_2_s may act by different mechanisms, leading to different cellular responses [14]. In contrast to the recombinant enzyme, natural hGX is glycosylated in mammalian cells and releases FFAs from intracellular membranes during its secretion [39]. To confirm that cell-derived hGX sPLA_2_ also has a positive effect on MDA-MB-231 cell growth and/or survival, we performed gain-of-function experiments by transiently expressing hGX sPLA_2_ and its catalytically inactive H48Q mutant. The expression and secretion of active hGX sPLA_2_ protein from transiently transfected cells was confirmed with a highly sensitive enzymatic assay using [^3^H]-labeled *E. coli* membranes [39]. Sub-nanomolar amounts of the enzyme ranging from 0.2 nM to 0.5 nM (corresponding to 10–40 ng/10^6^ cells) in the period 24–72 h after transfection were secreted in the extracellular medium from cells grown both in the presence and absence of serum (Additional file 2: Table S2). Most of the enzyme was secreted from the cells, since only about 1% of total hGX sPLA_2_ was detected in cell lysates 72 h after transfection (data not shown). Cells transiently expressing hGX sPLA_2_ displayed higher proliferation rates (Figure 1C) and were significantly more resistant to serum withdrawal-induced cell death (Figure 1D) than control cells. The mitogenic and the pro-survival effects were not observed in cells expressing the H48Q mutant of hGX sPLA_2_ and were completely abrogated by addition of the sPLA_2_ inhibitor varespladib to the culture media. It is important to emphasize that hGX sPLA_2_, both secreted from transfected MDA-MB-231 cells and the exogenously added recombinant protein (Additional file 1: Figure S1A), was biologically active at very low subnanomolar to nanomolar concentrations, which correspond to the putative endogenous concentrations of hGX sPLA_2_ suggested from the amounts determined in mouse tissues (0.3 nM in sera and 1–10 ng mGX/mg tissue protein; [40]). Thus, transiently expressed hGX sPLA_2_ is secreted from MDA-MB-231 cells in an active form and, through the products of its phospholipolytic activity, it stimulates cell proliferation and confers resistance to serum withdrawal-induced cell death.

Since sPLA_2_s may have opposing effects on cell growth in different cancer cells [17], we next asked whether hGX also prevents cell death in other breast cancer cells with different tumorigenic properties. Interestingly, hGX sPLA_2_ did not significantly affect the survival of the non-tumorigenic basal MCF-10A cells or of the weakly tumorigenic, estrogen receptor (ER) positive luminal MCF7 cells (Figure 1E). Further, it displayed a slight negative effect on the survival of the ER negative and HER2 positive SK-BR-3 cells. A weak, but statistically significant pro-survival effect, similar to that observed in the basal ER negative MDA-MB-231 cells, was observed in the weakly tumorigenic, ER positive luminal T-47D cells. Thus, hGX sPLA_2_ displays a differential ability to protect breast cancer cells from cell death, and of the cell lines tested, the effect was most prominent in the most tumorigenic and highly invasive triple negative MDA-MB-231 cell line.

### hGX sPLA_2_ prevents serum withdrawal-induced cell death by stimulating LD formation in MDA-MB-231 cells

**Figure 2:**
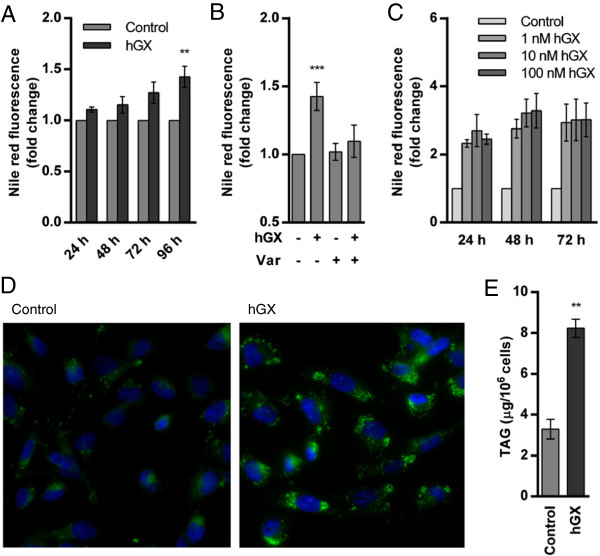
**hGX induces TAG synthesis and LD formation in MDA-MB-231 cells in an enzymatic activity-dependent manner. (A)** Quiescent MDA-MB-231 cells were treated with recombinant hGX (10 nM) in serum-free medium and neutral lipid accumulation was determined at indicated time points, using Nile red staining and flow cytometry. **(B)** Quiescent MDA-MB-231 cells were treated with hGX (10 nM) for 96 h in serum-free medium in the presence or absence of varespladib (Var; 50 μM) and stained with Nile red. **(C)** MDA-MB-231 cells were grown in complete culture medium in the presence of different concentrations of hGX and stained with Nile red at the indicated time points. **(D)** MDA-MB-231 cells were grown in complete culture medium in the presence of hGX (1 nM) for 48 h and TAG content was determined in cell lysates as described in Methods. The TAG content of hGX-treated cells was significantly higher than in that of control cells (**, P = 0.0086; Student’s *t*-test). **(E)** MDA-MB-231 cells were grown in complete culture medium in the presence of hGX (1 nM) for 48 h. The cells were fixed, stained with Nile red to visualize LDs (green) and DAPI to visualize nuclei (blue). The image shown is representative of two experiments. Values on the graphs are means ± SD of at least two independent experiments performed in duplicate. Results that are statistically significant over control samples are indicated (**, P < 0.01; ***, P < 0.001; one-way ANOVA with Bonferroni adjustment).

One of the most important observations from our flow cytometry analyses of MDA-MB-231 cells treated with hGX sPLA_2_ was the significant augmentation of cell granularity, inferred from the increase in the side scatter (SSC) parameter (Additional file 3: Figure S2). Such changes in cell morphology can be the result of extensive accumulation of neutral lipid in LDs, cytoplasmic organelles present in almost all cell types [31]. LDs not only store triglycerides and cholesterol esters to provide fuel and biosynthetic substrates, but can also prevent lipotoxicity and affect cell metabolism, growth and survival [3,31]. OA is a known inducer of LD formation in various cell types. It is also one of the most abundant FFAs incorporated into PC in cell membranes, including those of MDA-MB-231 cells [41], and is one of the major products of hGX sPLA_2_ activity on mammalian cells [12]. Importantly, exogenously added OA has been shown to induce LD accumulation in MDA-MB-231 cells, stimulate proliferation in serum-free media and prevent cell death induced by serum withdrawal [27,42]. Thus, we speculated that the pro-survival effect of hGX sPLA_2_ may be associated with LD formation stimulated by FFAs, including OA, released by hGX sPLA_2_ enzymatic hydrolysis of breast cancer cell membranes. In fact, recombinant hGX sPLA_2_ induced a gradual enzymatic activity-dependent increase in LD content in serum-starved MDA-MB-231 cells (Figures 2A and 2B) during the 96 h survival experiment (Figure 1B). The induction of LD formation was much more significant in proliferating MDA-MB-231 cells, as evidenced by flow cytometry and fluorescence microscopy analyses of Nile red stained cells (Figures 2C and 2D). The hGX-induced increase in neutral lipid staining in proliferating cells corresponded to the increase in TAG amount (Figure 2E), demonstrating that hGX stimulates TAG synthesis and incorporation into LDs. The higher level of hGX-induced LDs in proliferating cells than in serum-starved, quiescent cells is in line with the predominant anabolic metabolism [1,4] and the higher availability of phospholipid substrates for hGX sPLA_2_ in proliferating cells, including cell membranes and serum lipoproteins [12,43]. Most of the LD formation in proliferating MDA-MB-231 cells occurred within the first 24 h of treatment and the level of accumulated LDs reached maximal values after 48 h of incubation with the enzyme (Figure 2C). This could explain the modest and saturable positive effect of hGX on MDA-MB-231 cell proliferation in serum-containing media (Additional file 1: Figure S1A) and its augmentation on serum-deprived cells (Figure 1A).

**Figure 3:**
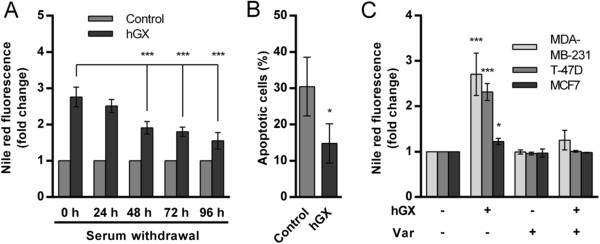
**LDs formed by the action of hGX convey a survival advantage to MDA-MB-231 cells during serum deprivation. (A)** MDA-MB-231 cells were grown in complete culture medium in the presence of hGX for 48 h (1 nM). The cells were washed, sPLA_2_-free and serum-free medium containing 0.02% BSA was added, and the cells incubated further without medium renewal. Neutral lipid content was measured by Nile red staining at the indicated time points during starvation. Note that LD content gradually decreased during serum-starvation and that cells that contained pre-formed hGX-induced LDs displayed an increased survival rate after 96 h of serum-starvation, as determined by the TMRM/YO-PRO-1 apoptosis assay **(B)**. **(C)** Breast cancer cell lines were grown in complete culture medium with hGX (10 nM) in the presence or absence of varespladib (Var; 50 μM) for 24 h and cellular LD content was measured by Nile red staining. Values on the graphs are means ± SD of at least two experiments performed in duplicate and results that are statistically significant over control samples are indicated (*, P < 0.05; ***, P < 0.001; one-way ANOVA with Bonferroni adjustment).

The energy and building blocks stored in LDs during growth in serum may be used gradually by the cell to support cell growth and survival depending on its needs [3]. It was thus reasonable to investigate whether hGX-induced LDs accumulated in proliferating cells would provide a survival advantage to the cells on serum withdrawal and removal of the enzyme. To this end, MDA-MB-231 cells were treated with hGX sPLA_2_ for 48 h in the presence of serum (LD accumulation phase), and the lipid content and cell survival then measured during the subsequent 96 h period of serum-free incubation in the absence of sPLA_2_ and without medium renewal (LD consumption phase). The LDs formed by the action of hGX sPLA_2_ were consumed during the subsequent serum starvation phase (Figure 3A) and, importantly, the cells, which now contained large amounts of pre-formed LDs, displayed a remarkable survival rate even in the absence of the enzyme (Figure 3B).

To strengthen the proposal that LDs are responsible for the effects of hGX on breast cancer cell survival, we next investigated whether the differential ability of hGX sPLA_2_ to stimulate cell survival in different breast cancer cells (Figure 1E) corresponds to its LD-inducing potency in a particular cell line. While hGX was observed to induce a very significant increase in LD accumulation in MDA-MB-231 and T-47D cells (Figure 3C), which were both protected from cell death by hGX during starvation (Figure 1E), it induced only a modest increase in LD formation in MCF7 cells and did not affect their survival upon serum withdrawal. A lipogenic phenotype has been suggested for MDA-MB-231 cells, but not for MCF7 cells, indicating that the ability of hGX sPLA_2_ to induce LDs is dependent on the capacity of a particular cell line to synthesize and store large amounts of TAGs [42,44]. These results strongly support the proposal that the LDs formed due to the enzymatic action of hGX sPLA_2_, in both rapidly proliferating and quiescent cells, are responsible for the increased survival of breast cancer cells during serum deprivation. Clearly, rather than conferring an immediate proliferative advantage to the cells grown under optimal conditions, hGX-induced changes in lipid accumulation are associated with a pro-survival, anti-apoptotic mechanism that is triggered under conditions of nutrient and growth factor limitation.

### The ability of hGX sPLA_2_ to hydrolyze PC-rich membranes is important for the stimulation of LD formation and its pro-survival action

**Figure 4:**
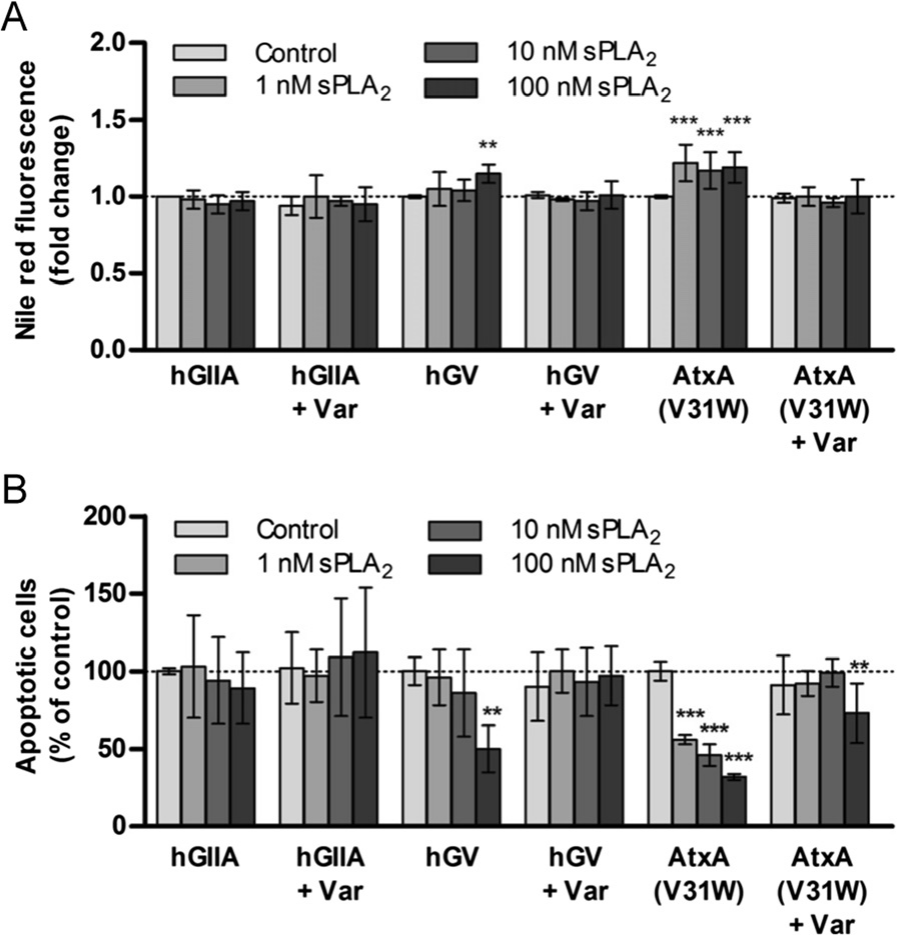
**The effects of hGX are reproduced by other PC-hydrolyzing sPLA**_**2**_**s. (A)** Quiescent MDA-MB-231 cells were treated with the indicated concentrations of recombinant hGIIA, hGV and AtxA(V31W) in combination with varespladib (Var; 50 μM) in serum-free medium for 96 h. Cellular neutral lipid content was then determined by Nile red staining **(A)** and the percentage of apoptotic cells within the population was measured in parallel samples by the TMRM/YO-PRO-1 assay **(B)**. The hGV and AtxA(V31W) sPLA_2_s, similar to hGX in their ability to hydrolyze PC-rich membranes [13,46], induced LD formation and prolonged cell survival in an enzymatic activity-dependent manner, while hGIIA did not induce LD formation or prolong survival. Values on the graphs are means ± SD of three experiments performed in duplicate. Results that are statistically significant over control samples are indicated (**, P < 0.01; ***, P < 0.001; one-way ANOVA with Bonferroni adjustment).

The different human and venom sPLA_2_s exhibit specific and subtle substrate preferences towards phospholipid head groups and acyl chains at the *sn*-2 position, which result from their distinct membrane-binding affinities to various phospholipid interfaces and also from subtle differences in their active sites [13,45,46]. Since hGX sPLA_2_ is known for its high binding affinity and efficient hydrolysis of PC-rich cell membranes [12,45], we assumed that its potency in inducing LD formation and cell survival in MDA-MB-231 cells could depend on this property. Accordingly, two other sPLA_2_s with similarly high affinities for PC-rich surfaces coupled with the ability to release FFAs from mammalian cells, the human group V (hGV) sPLA_2_[13] and the ammodytoxin A (AtxA) V31W mutant, a neurotoxic snake venom sPLA_2_[46], induced significant LD formation and protected MDA-MB-231 cells from starvation-induced cell death in an enzymatic activity-dependent manner (Figures 4A and 4B). In contrast, the human group IIA enzyme, which does not bind well to or hydrolyze the PC-rich plasma membranes of mammalian cells [45], did not induce LD formation or support cell survival. Thus, the ability of an sPLA_2_ to bind with high affinity and hydrolyze cell membrane phospholipids is essential for the induction of LD formation and for breast cancer cell survival. These results provide an additional proof that the hydrolytic products released from MDA-MB-231 cell membranes by hGX sPLA_2_ are responsible for the formation of LDs, which in turn confer resistance to serum withdrawal-induced apoptosis.

### Oleic acid is an important mediator of hGX-induced LD formation and cell survival in MDA-MB-231 breast cancer cells

**Figure 5:**
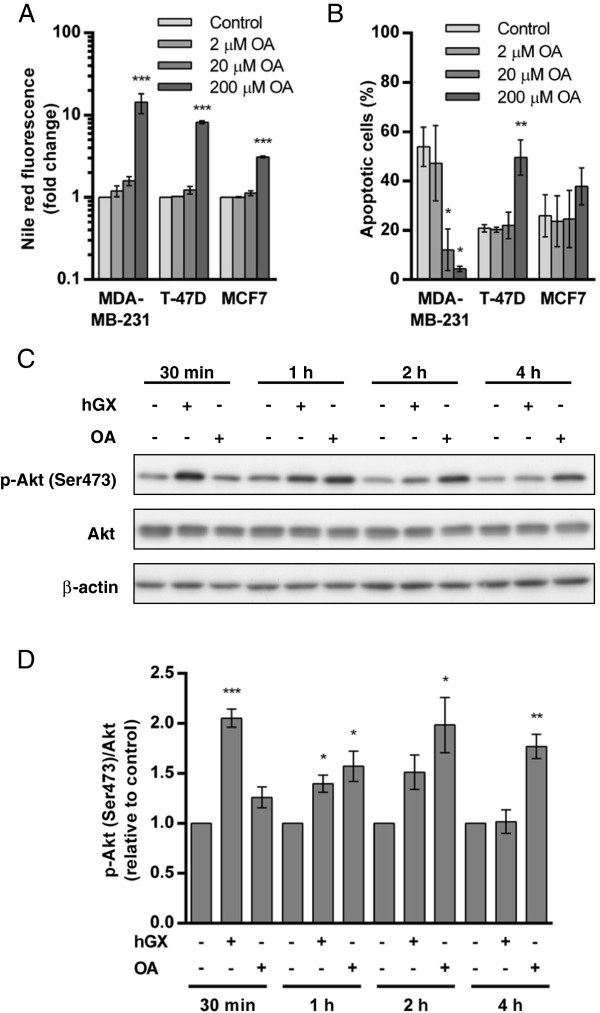
**Recombinant hGX sPLA**_**2 **_**and OA activate the pro-survival Akt kinase and display similar effects in breast cancer cells. (A, B)** MDA-MB-231, T-47D and MCF7 cell lines were grown in serum-free medium for 96 h in the presence of the indicated concentrations of OA complexed with 0.5% FAF BSA. LD content was determined by Nile red staining **(A)** and cell death was assessed in parallel samples using the TMRM/YO-PRO-1 apoptosis assay **(B)**. **(C)** MDA-MB-231 cells were made quiescent by 48 h serum starvation, then washed and incubated with hGX (10 nM) or OA (100 μM) in serum-free medium for the times indicated. Cell lysates were analyzed for the presence of Ser473-phosphorylated Akt (p-Akt) kinase, total Akt protein and β-actin loading control by immunoblotting. The amount of p-Akt was normalized to total Akt protein and was quantified relative to untreated controls at each specific time point **(D)**. Values on the graphs are means ± SD of at least two experiments performed in duplicate and results that are statistically significant over control samples are indicated (*, P < 0.05; **, P < 0.01; ***, P < 0.001; one-way ANOVA with Bonferroni adjustment).

It has been shown that exogenously added OA and other monounsaturated FAs induce LD formation and stimulate the growth and survival of various cells, including breast cancer cells [27,29]. Furthermore, short-term exposure of MDA-MB-231 cells to high micromolar concentrations of exogenous OA leads to accumulation of TAG, increased lipolysis and a long-term resistance to serum withdrawal-induced apoptosis [42]. The patterns of lipid accumulation (Figure 3C) and of cell survival changes (Figure 1E) induced by hGX sPLA_2_ are very similar to that reported previously where exogenous OA stimulated TAG synthesis and protected MDA-MB-231 and T-47D, but not MCF7 and MCF-10A, cells from serum starvation-induced cell death [42]. This suggests that the effects of hGX sPLA_2_ may be mediated by OA. We next confirmed that OA is readily released from the membranes of adherent MDA-MB-231 cells by hGX sPLA_2_ (Additional file 4: Figure S3) and demonstrated that exogenously added OA and recombinant hGX sPLA_2_ display similar abilities to induce LD formation in the three cell lines tested (Figure 5A). In contrast to hGX sPLA_2_, however, OA prevented cell death only in MDA-MB-231 cells (Figure 5B). This lack of a pro-survival effect of OA in T-47D cells, despite their relatively high levels of LDs, indicates that other sPLA_2_ hydrolytic products are involved in the anti-apoptotic activity of hGX, in particular in T-47D cells. Since Hardy *et al.* have shown [27,28,47] that OA stimulates MDA-MB-231 cell proliferation by activating the phosphatidylinositol 3-kinase (PI3K)/Akt pathway, we asked whether hGX also activates Akt kinase. In fact, both hGX and OA increased the level of Akt phosphorylation at Ser-473 in starved MDA-MB-231 cells (Figure 5C). Interestingly, the dynamics of Akt activation were different with hGX maximally activating Akt after 30 min, while OA was less effective and reached a similar level only after 2 h of incubation (Figure 5D). Nevertheless, this supports the idea that OA mediates, at least in part, the effects of hGX sPLA_2_ and that activated Akt supports the anti-apoptotic and metabolic alterations caused by hGX sPLA_2_[1]. Clearly, OA, as one of the most abundant FAs released from cell membranes by the action of hGX, may be one of the dominant lipid mediators of the effects of hGX on MDA-MB-231 cells by being the major FFA feeding the pathway of TAG synthesis and LD formation and also triggering pro-survival signaling.

We have shown previously that hGX sPLA_2_ releases a complex mixture of mitogenic products from colon cancer cells, including AA, OA and linoleic acid, lysophosphatidic acid (LPA), and eicosanoids [26]. To assess the involvement in the effects of hGX sPLA_2_ of several signaling pathways activated by these products [23,47], we tested a range of pharmacological inhibitors for their ability to interfere with hGX-induced LD formation and survival. The cPLA_2_α inhibitors AZ-1 [48] and pyrrolidine-2 did not suppress the stimulating effect of hGX on LD formation in proliferating cells (Additional file 5: Figure S4A). Pyrrolidine-2 increased both basal and hGX-induced LD formation and was slightly toxic to starving MDA-MB-231 cells, but did not affect the pro-survival activity of hGX (Additional file 5: Figure S4B). The non-selective cyclooxygenase (COX) inhibitor indomethacin only slightly inhibited LD formation, but did not affect hGX-induced cell survival (Additional file 4: Figures S3A and S3B). The mammalian target of rapamycin (mTOR) pathway may be activated by PI3K/Akt signaling, as well as by AA [49], to alter lipid metabolism and stimulate anabolic growth in breast cancer cells [50]. The mTOR inhibitor rapamycin marginally reduced hGX-induced LD formation (Additional file 5: Figure S4A), indicating that mTOR may participate in, but is not critical for, hGX-induced LD formation. Finally, results with two inhibitors of the autotaxin (ATX)-LPA axis, S32826 and BrP-LPA [51,52] (Additional file 5: Figure S4A), indicate that LPA signaling is not necessary for the effects of hGX. These results, therefore, suggest that, while cPLA_2_α activity and the ATX-LPA axis are not involved in the effects of hGX, COX-mediated AA-metabolism and mTOR signaling may contribute to, but are not critical for, the LD-promoting and pro-survival activities of hGX in MDA-MB-231 cells. These results further strengthen the suggestion that, of the various hydrolytic products released by hGX sPLA_2_ from cell membranes, OA plays a very important role in LD formation, lipid metabolism alterations and pro-survival signaling in MDA-MB-231 breast cancer cells.

### Etomoxir suppresses hGX-induced LD accumulation and cell survival in serum-starved MDA-MB-231 cells

**Figure 6:**
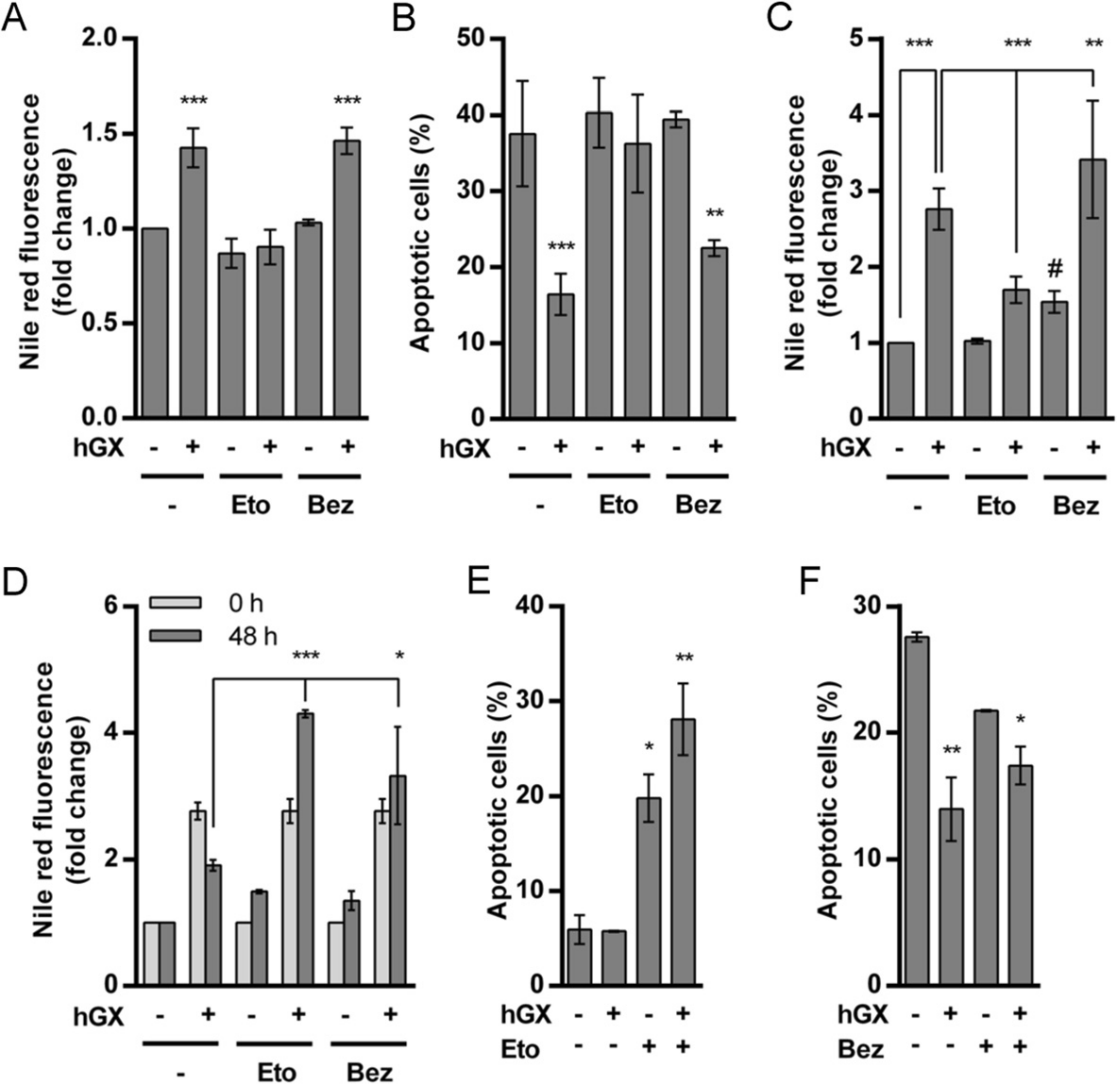
**Etomoxir, an inhibitor of β-oxidation, suppresses hGX-induced LD biogenesis and cell survival. (A, B)** Quiescent MDA-MB-231 cells were treated with hGX (10 nM) in serum-free medium containing 0.02% FAF BSA for 96 h in the presence or absence of etomoxir (Eto; 20 μM) and bezafibrate (Bez; 500 μM). Etomoxir, but not bezafibrate, prevented both hGX-induced LD formation **(A)** and cell survival **(B)**. **(C)** MDA-MB-231 cells were grown in complete medium for 24 h and then treated with hGX (1 nM) in complete medium for 48 h in the presence or absence of either etomoxir (20 μM) or bezafibrate (500 μM). Etomoxir significantly reduced the level of hGX-induced LDs in proliferating cells, while bezafibrate stimulated control LD formation (#, P < 0.05) and supported hGX-induced LD formation. **(D, E)** MDA-MB-231 cells were pre-treated with hGX (10 nM) for 48 h in complete medium to form LDs; the cells were then washed (0 h) and incubated in serum-free and hGX-free medium for an additional 48 h in the presence or absence of either etomoxir (250 μM) or bezafibrate (500 μM). The levels of LDs remaining after serum deprivation (48 h) were compared with those at the beginning of starvation (0 h). Both agents significantly prevented LD breakdown during starvation **(D)**. **(E, F)** MDA-MB-231 cells were treated as described in **(D)** and the percentage of apoptotic cells determined following 48 h **(E)** or 96 h **(F)** serum deprivation. Etomoxir (250 μM) alone significantly increased the percentage of apoptotic cells in samples with preformed LDs, and also in hGX-untreated cells **(E)**. Values on the graphs are means ± SD of at least two experiments performed in duplicate. Results that are statistically significant are indicated (*, P < 0.05; **, P < 0.01; ***, P < 0.001; one-way ANOVA with Bonferroni adjustment).

It has been shown recently that β-oxidation contributes to tumorigenesis [35,53] and may protect cancer cells from starvation-induced cell death [6,8-10]. Additionally, it has been suggested that it complements LD accumulation as a mechanism of preventing lipotoxicity in cells exposed to high levels of exogenous FAs [31]. We therefore hypothesized that β-oxidation could be important for cell survival in hGX-treated cells and that it may contribute through either one or both of the following mechanisms: β-oxidation of FFAs released by sPLA_2_ membrane hydrolysis and/or liberated from LDs through lipolysis. We therefore sought to determine whether pharmacological modulators of β-oxidation would affect the positive effects of hGX on MDA-MB-231 cell survival and LD formation. Etomoxir is an irreversible inhibitor of carnitine palmitoyltransferase 1 (CPT1), the rate-limiting enzyme in β-oxidation that transports activated FAs across the mitochondrial membrane [54]. It effectively suppresses β-oxidation in various cells and tissues [6,9,54], including MDA-MB-231 breast cancer cells [28]. Remarkably, when MDA-MB-231 cells were incubated for 96 h in serum-free medium in the presence of low, non-toxic concentrations of etomoxir (20 μM), the increase in LD accumulation (Figure 6A) and the anti-apoptotic activity (Figure 6B and Additional file 6: Figure S5) of hGX sPLA_2_ were completely abolished. This strengthens the above conclusions that the prolonged survival of serum-deprived MDA-MB-231 cells is dependent on the hGX-induced LDs. Surprisingly, without affecting the levels of neutral lipids in control cells, etomoxir significantly reduced accumulation of LDs in well-fed, proliferating MDA-MB-231 cells treated with hGX for 48 h (Figure 6C), when β-oxidation is expected to be minimal [1]. This was unexpected, since etomoxir treatment typically leads to a compensatory increase in TAG accumulation [54], presumably reflecting an attempt of the cell to minimize the lipotoxicity of accumulating FFAs in the cytosol [31]. This suggests that etomoxir may also suppress the pro-survival action of hGX in starved cells by reducing hGX-induced LD formation. To confirm that β-oxidation is critical for cell survival enabled by hGX-induced LDs, we tested the ability of etomoxir to alter the survival of serum-deprived MDA-MB-231 cells that actively consume pre-formed LDs (Figure 3A) and in which β-oxidation is presumably highly active. LD breakdown was effectively blocked by etomoxir (Figure 6D), even leading to increased levels of neutral lipids, indicating that, by blocking β-oxidation, etomoxir also suppressed LD lipolysis and that any additional FFAs are channeled towards TAG synthesis [55]. Importantly, etomoxir not only abolished the positive effect of LDs on cell survival, but also induced cell death in both control cells and in cells with pre-formed droplets (Figure 6E), strongly suggesting that β-oxidation is necessary for cell survival during starvation. Thus, while non-toxic concentrations of etomoxir suppressed hGX-induced LD formation and cell survival in quiescent cells, higher concentrations of the inhibitor prevented LD consumption and abolished their anti-apoptotic effect when added to cells with pre-formed LDs.

On the other hand, bezafibrate, a pan-peroxisome proliferator-activated receptor (PPAR) agonist and an activator of mitochondrial biogenesis and β-oxidation [3,6,56], did not affect hGX-induced accumulation of LDs or cell survival in starved cells (Figures 6A and 6B), but caused a slight increase in both basal and hGX-induced LD accumulation levels in proliferating cells (Figure 6C). During the LD consumption phase in cells with pre-formed LDs, bezafibrate suppressed LD breakdown, even inducing further accumulation of LDs in both control and hGX-treated cells, but did not prevent the pro-survival effect of the LDs (Figures 6D and 6F). Accordingly, besides stimulating mitochondrial biogenesis and β-oxidation, including the expression of CPT1 [54], PPARα activation has also been shown to induce TAG accumulation [57]. Thus, bezafibrate stimulates LD accumulation and effectively prevents net LD breakdown in starving MDA-MB-231 cells, but does not block hGX-induced LD formation or cell survival, most probably due to its ability to stimulate β-oxidation as well. This is in line with the suggestion that active β-oxidation contributes to LD formation and is necessary for cell survival in hGX-treated MDA-MB-231 cells. Collectively, the results of these experiments using pharmacological modulators of FA metabolism confirm that the pro-survival effect of hGX in MDA-MB-231 cells depends on its ability to stimulate LD formation. They also support a hypothesis that β-oxidation contributes to the process of hGX-induced LD biogenesis in MDA-MB-231 cells, regardless of their metabolic and proliferative status, and is critical for the effect of hGX-induced LDs on cell survival during starvation.

### hGX sPLA_2_ alters the expression of major lipid metabolism genes

**Figure 7:**
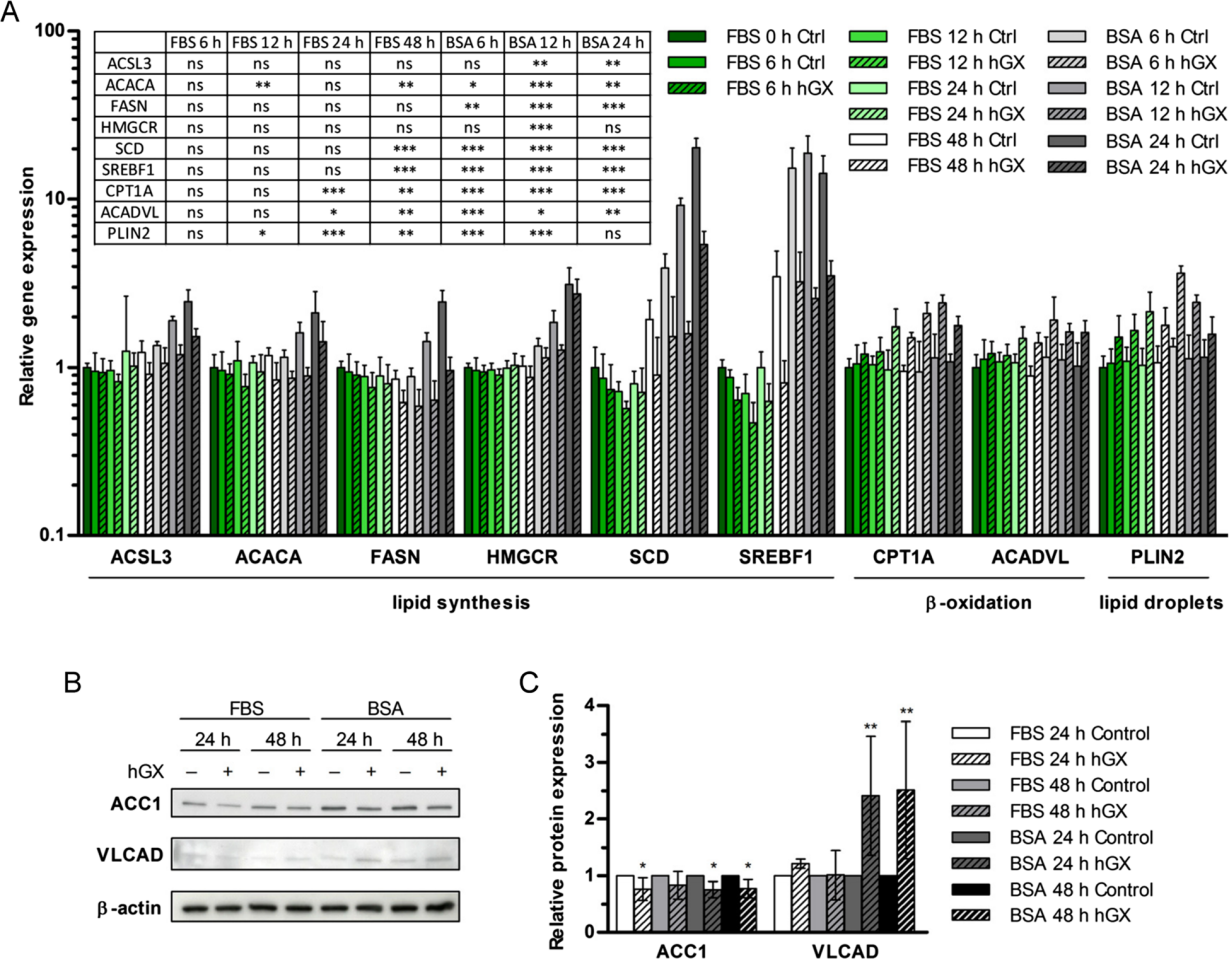
**hGX alters the expression levels of genes involved in lipid metabolism.** MDA-MB-231 cells were grown in complete culture medium in the presence of hGX (1 nM) for 48 h. The medium was removed and cells were washed and incubated for an additional 24 h in serum-free and hGX-free medium containing 0.02% FAF BSA. Cell lysates were prepared at the time points indicated and the relative expression levels of genes involved in lipid metabolism were determined by means of qPCR **(A)**. The relative protein expression levels of ACC1 and VLCAD were analyzed by immunoblotting **(B)** at the indicated time-points. **(C)** Protein band intensities were analyzed by densitometry and normalized to loading control (β-actin) values at each time-point. Values on the graphs are means ± SD of three experiments performed in duplicate and results that are statistically significant over control samples are indicated (*, P < 0.05; **, P < 0.01; ***, P < 0.001; one-way ANOVA with Bonferroni adjustment).

Since our results suggest that the pro-survival effect of hGX in MDA-MB-231 cells depends on neutral lipid accumulation and β-oxidation, we sought whether hGX may affect the expression of major lipid metabolism and LD-associated genes. Using qPCR, we analyzed the expression of a set of 38 selected genes involved in FA activation (*ACSL1, ACSL3, ACSL4, ACSL5, ACSL6*), FA oxidation (*CPT1A, ACADL, ACADVL, HADHA, HADHB*) and synthesis (*ACACA, ACACB, FASN, SCD*), TAG synthesis (*GPAM, GPAT2, AGPAT6, AGPAT9, DGAT1, DGAT2*) and lipolysis (*PNPLA2, MGLL*), cholesterol metabolism (*HMGCR, SOAT1, CAV1*), LD-associated proteins (*PLIN1, PLIN2, PLIN3, PLIN4, PLIN5, CIDEB*), lipid metabolism transcription factors (*SREBF1, PPARA, PPARG, RXRB*), lysophosphatidylcholine (LPC) esterification (*LPCAT1, LPCAT2*) and FA uptake (*SCL27A1*). No alterations in the expression levels of these genes were found in serum-deprived MDA-MB-231 cells treated with hGX for 96 h (data not shown). However, there were significant changes in the expression of several genes (Figure 7A) when MDA-MB-231 cells were treated with hGX for 48 h in the presence of serum to induce maximal LD biogenesis (Figure 2C), then serum-deprived for 24 h in the absence of hGX to allow high levels of LD lipolysis (Figure 3A). Eight time-points were analyzed to search for possible correlations between the time-course of net LD accumulation in proliferating cells and LD consumption in starved cells with changes in gene expression. A significant decrease was detected in the expression of genes for the lipogenic transcription factor sterol regulatory element-binding protein 1 (SREBP-1) and the key FA synthesis enzymes ACC1, FAS and stearoyl-CoA desaturase 1 (SCD-1) [2,4,29] (Figure 7A). Small but significant decreases were also detected for the long-chain acyl-CoA synthetase 3 (ACSL3) and the hydroxymethylglutaryl-coenzyme A reductase (HMGCR) enzymes. Interestingly, changes in the expression of most of the lipogenic genes were first observed at the 48 h time-point, when MDA-MB-231 cells had typically accumulated their maximal level of LDs, but were greatest 12 h after the cells were switched to serum-free media. The basal expression of the genes encoding for FAS, SCD-1 and SREBP-1 was elevated at these time-points, suggesting that hGX acts on MDA-MB-231 cells to suppress their induction, most probably due to a rising need for *de novo* lipid synthesis. On the other hand, there was a significant increase in the mRNA levels of two key β-oxidation enzymes, CPT1A and very long-chain acyl-CoA dehydrogenase (VLCAD) – the first enzyme in the β-oxidation cycle [58]. In contrast to the lipogenic genes, the expression of the two β-oxidation genes was augmented by hGX after only 24 h of cell growth and was further increased at the beginning of the starvation period. Interestingly, there was no alteration in the basal expression levels of these genes, suggesting that their expression is not regulated by serum deprivation. Furthermore, the mRNA level of the LD-coating protein perilipin 2 (PLIN2; Figure 7A), that promotes LD formation and regulates lipolysis in different cells [3,57,59], was also higher in hGX-treated cells. Its mRNA levels were significantly elevated after only 12 h of incubation of proliferating MDA-MB-231 cells with hGX; they reached maximal levels 6 h after serum withdrawal and decreased steadily over the final 18 h of starvation, suggesting a correlation between the amount of LDs and perilipin 2 mRNA levels in MDA-MB-231 cells. The hGX-induced alterations in gene expression were confirmed at the protein level for the first enzymes in FA synthesis and β-oxidation, ACC1 and VLCAD, respectively, corroborating the qPCR results (Figures 7B and 7C). Collectively, these results strongly suggest that proliferating MDA-MB-231 cells respond to the products of hGX phospholipolysis first by up-regulating perilipin 2, which supports LD formation [3], followed very closely by an increase in the expression of the major β-oxidation enzymes, CPT1 and VLCAD, suggesting an augmentation of the rates of β-oxidation [60]. When the amount of accumulated LDs reaches its maximal levels (Figure 2C), and after serum withdrawal, when LDs are rapidly consumed (Figure 3A), the induction of the expression of lipogenic genes, in particular the ones encoding SREBP-1, ACC1, FAS and SCD, is significantly repressed, while expression of the key β-oxidation enzymes, CPT1 and VLCAD, reaches maximal levels. Clearly, the hGX-induced LD accumulation in MDA-MB-231 cells is accompanied by significant changes in the expression of major lipid metabolism genes, indicative of an increase in β-oxidation and LD formation, as well as a reciprocal decrease in *de novo* FA and cholesterol synthesis.

### hGX-induced LD formation is associated with activation of AMPK

**Figure 8:**
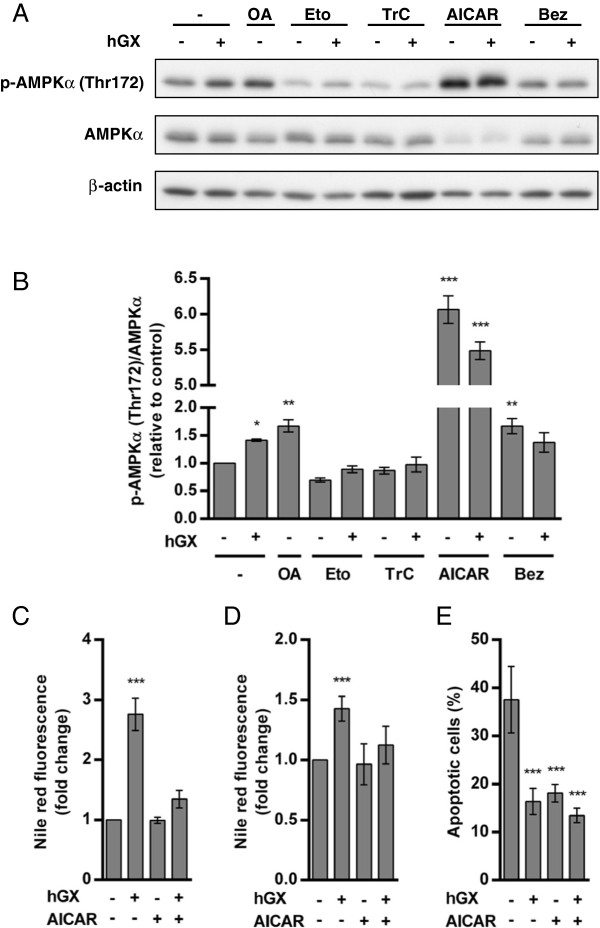
**hGX-induced LD accumulation is associated with activation of AMPKα. (A)** MDA-MB-231 cells were treated with OA (100 μM) and hGX (1 nM) in complete culture medium for 48 h in the presence of etomoxir (Eto; 20 μM), triacsin C (TrC; 2 μM), AICAR (500 μM) or bezafibrate (Bez; 500 μM). Free OA was incubated in complete culture medium for 1 h before addition to the cells. Cell lysates were analyzed for the presence of Thr172-phosphorylated AMPKα (p-AMPKα), total AMPKα and β-actin loading control by immunoblotting and densitometry. The amounts of p-AMPKα obtained from three separate experiments were normalized to total AMPKα protein levels and quantified relative to untreated controls **(B)**. **(C)** Cells were treated as in **(A)**. Prolonged incubation (48 h) of proliferating MDA-MB-231 cells with AICAR (500 μM) abolished the hGX-induced (1 nM) LD formation. Cellular LD content was determined by Nile red staining. **(D, E)** Serum-starved MDA-MB-231 cells were treated with hGX (10 nM) in serum-free medium containing 0.02% FAF BSA for 96 h in the presence or absence of AICAR (500 μM). After 96 h, cellular LD content was determined by Nile red staining, showing that AICAR prevented hGX-induced LD formation **(D)**. Cell survival was assessed with the TMRM/YO-PRO-1 apoptosis assay, indicating that AICAR alone has a pro-survival effect in MDA-MB-231 cells, thus effectively masking the positive effect exerted by hGX. Values on the graphs are means ± SD of at least two experiments performed in duplicate and results that are statistically significant over control samples are indicated (*, P < 0.05; **, P < 0.01; ***, P < 0.001; one-way ANOVA with Bonferroni adjustment).

AMPK is a central metabolic sensor and reciprocal regulator of cellular metabolism. It blocks anabolic and activates energy producing processes in response to low energy states of the cell [61]. Activation of AMPK increases β-oxidation and TAG lipolysis [62] and inhibits FA and TAG synthesis [61,63]. Importantly, AMPK increases cancer cell growth and survival during energy stress by altering FA metabolism [6,8,60]. An increase in the amount of Thr172-phosphorylated AMPKα (p-AMPKα) in proliferating MDA-MB-231 cells was observed after 48 h of growth in the presence of recombinant hGX sPLA_2_ or exogenous OA (Figures 8A and 8B). This showed that the effects of hGX on LD formation and cell survival are associated with the activation of AMPK. In line with their ability to suppress hGX-induced LD formation (Figures 6C and Additional file 5: Figure S4A), etomoxir and the non-selective ACS inhibitor triacsin C prevented the increase in p-AMPKα levels induced by hGX. Bezafibrate, on the other hand, increased the basal level of activated AMPK and hGX did not further elevate p-AMPKα levels, in keeping with its effects on LD formation (Figure 6C). These results suggest that the levels of p-AMPKα correlate with the amount of hGX-induced LDs. In support of this, LD accumulation reached peak levels after 48 h in hGX-treated proliferating MDA-MB-231 cells (Figure 2C), suggesting that the increase in AMPK activation may be a consequence of extensive TAG synthesis and LD formation [29]. These results therefore point to the effects of hGX on LD formation and cell survival being associated with a regulatory mechanism involving AMPK. Further, timely activation of AMPK, leading to blockade of LD formation may be crucial for preventing excessive energy consumption in rapidly proliferating MDA-MB-231 cells treated with hGX. To substantiate this view, we asked whether prolonged activation of AMPK would prevent the LD formation induced by hGX. Activating AMPK with the AMP-analog 5-aminoimidazole-4-carboxamide ribonucleoside (AICAR) [6] (Figures 8A and 8B) completely abolished hGX-induced LD formation in both proliferating (Figure 8C) and in starved MDA-MB-231 cells (Figure 8D), indicating that AMPK activation indeed blocks hGX-induced LD biogenesis. This is in line with the complete blockade of lipid synthesis caused by AICAR in MDA-MB-231 cells [64]. It further raised the question as to whether the suppression of LD biogenesis by AICAR would abolish the positive effect of hGX on cancer cell survival during serum deprivation. We found that prolonged treatments with AICAR reduced the basal level of dying cells in the starving MDA-MB-231 cell population to a level similar to that observed with hGX itself (Figure 8E), thus effectively masking the positive effect of hGX. The effect of AICAR accords with the recently reported role for AMPK in enabling cancer cell survival during energy stress by suppressing lipogenesis and activating β-oxidation [6,8]. It is therefore also consistent with the proposed importance of hGX-induced alterations in FA metabolism for the survival of hGX-treated MDA-MB-231 cells. Thus, prolonged activation of AMPK by AICAR in MDA-MB-231 cells prevents hGX-induced lipid accumulation by blocking LD biogenesis in both proliferating and starved cells, suggesting that the role of AMPK may indeed be to suppress TAG synthesis and LD formation in hGX-treated cells.

## Discussion

We have demonstrated here that hGX sPLA_2_-mediated phospholipid hydrolysis induces LD formation and alters lipid metabolism in triple negative breast cancer cells, stimulating their proliferation and prolonging cell survival during serum deprivation. Several mammalian sPLA_2_s have been shown to stimulate cell proliferation in cancer cells [18,19,22,25]. An anti-apoptotic role in growth factor-deprived cells has been shown for the group IIA in kidney fibroblasts [65], and for the group III and X sPLA_2_s in neuronal cultures [66,67]. The enzymatic activity-dependent mechanisms reported [18,22,65] have usually been associated with the pleiotropic actions of AA-derived eicosanoids [25] and rarely with lysophospholipids [26,66]. The involvement of signaling pathways triggered by sPLA_2_ receptors on the cell surface has been also suggested in some cases [68,69]. However, the exact molecular mechanisms involved in the effects of sPLA_2_ on cell fate have not been elucidated nor has the relevance of these activities in mammalian pathophysiology been delineated [14]. This is not surprising given the differential tissue expression patterns of sPLA_2_s, the vast variety of extracellular target membranes and the plethora of bioactive products that are released from cell membranes in response to the action of sPLA_2_s [12,26]. hGX sPLA_2_ has been shown to induce colon cancer cell proliferation by releasing a complex mixture of mitogenic FAs, lysophospholipids and eicosanoids, but, surprisingly, the proliferative effects of hGX sPLA_2_ were not dependent on the mitogenic activity of AA-derived prostaglandin or LPC-derived LPA signaling [26]. Information about the possible involvement of sPLA_2_s in the modulation of cellular metabolism, rather than direct bioactive lipid signaling, is only beginning to emerge [15]. Here we show that hGX sPLA_2_ acts through the products of its hydrolysis and induces significant alterations in fatty acid metabolism and storage in breast cancer cells. These changes result primarily in prevention of serum withdrawal-induced cell death rather than in stimulation of cell proliferation. The previously reported mitogenic effects of sPLA_2_s in colon cancer and other cells were also modest [18,19,22,25,26], suggesting that the positive effects of sPLA_2_s on cell proliferation could, at least in some of these studies, be in fact a consequence of underlying changes in basic lipid metabolism and a pro-survival action, which is most evident under stressful conditions for the cell.

Several lines of evidence demonstrate that the effects of hGX sPLA_2_ on breast cancer cells are dependent on its enzymatic activity. First, the effects of recombinant hGX sPLA_2_ on MDA-MB-231 cell proliferation (Figure 1A), cell survival (Figure 1B) and LD formation (Figure 2B) were prevented by the potent sPLA_2_ inhibitor varespladib. Secondly, it also prevented the effects of ectopically expressed hGX sPLA_2_. The transient expression of its catalytically impaired H48Q mutant did not affect MDA-MB-231 cell proliferation or survival upon serum withdrawal (Figures 1C and 1D). Further, the fact that varespladib, an inhibitor with low cell membrane permeability, completely prevents the actions of exogenous and ectopically expressed hGX argues for an extracellular action of the enzyme. Thirdly, while the potency of the recombinant mGX sPLA_2_ to stimulate cell proliferation was similar to that of the human enzyme, its H48Q mutant did not induce a significant change in MDA-MB-231 cell proliferation rate (Additional file 1: Figure S1B). Fourthly, two other sPLA_2_ enzymes, hGV sPLA_2_[13] and a neurotoxic snake venom sPLA_2_, AtxA(V31W) [46], each with high activity on mammalian cell membranes, prevented cell death and induced LD formation in a varespladib sensitive manner. The hGV sPLA_2_ enzyme was less effective than hGX in inducing these cellular effects, which is consistent with previous results showing a better ability of the latter enzyme to act on plasma membranes of mammalian cells and release free FAs, such as arachidonic acid [13,45]. In contrast, the hGIIA sPLA_2_ enzyme, known for its inability to bind to PC-rich membranes and act on intact mammalian cells [45], was unable to induce LD formation or prevent MDA-MB-231 cell death. These facts lead to the conclusion that the LD formation and prevention of cell death induced by hGX in MDA-MB-231 cells are dependent on the ability of hGX to bind to and hydrolyze phospholipid membranes.

Numerous studies have shown that hGX sPLA_2_ is the most potent of the mammalian sPLA_2_ enzymes in hydrolyzing PC-rich phospholipid vesicles, plasma membranes and lipoprotein particles, thus releasing large amounts of lysophospholipids and unsaturated FAs, including oleic, linoleic and arachidonic acids [12,13,26,43]. Thus, lipoprotein particles are an important target for hGX sPLA_2_ hydrolysis during cell culture in the presence of serum; however, the major source of lipid for hGX-induced LD generation in serum-deprived cells must be the cell membranes of MDA-MB-231 cells. hGX sPLA_2_ may act directly on the plasma membrane of the cells and/or on microvesicles being actively released and recycled by MDA-MB-231 cells [70], as well as on apoptotic cells during starvation [71]. Regardless of the source of lipid, the results of this study indicate that of the products typically released upon hGX sPLA_2_ membrane hydrolysis OA is largely responsible for the metabolic and signaling alterations that support its pro-tumorigenic effects. Exogenous OA is known to induce a PI3K/Akt-dependent proliferation, stimulate LD formation and prevent serum withdrawal-induced apoptosis in MDA-MB-231 cells [27,28,42,47]. hGX sPLA_2_ is shown in the present work to stimulate cell proliferation and increase the survival of serum-deprived MDA-MB-231 cells (Figures 1A and 5C and 5D). Further, exogenous hGX and OA are both shown to activate AMPK in proliferating cells (Figures 8A and 8B), strongly suggesting that OA is one of the major mediators of the pro-tumorigenic effects of hGX. Importantly, the effects of OA are not restricted to breast cancer cells, since there is ample evidence that OA feeds into the TAG synthesis pathway and stimulates LD formation, cell growth and survival in different non-adipose cells, even channeling saturated FAs to TAGs to prevent their apoptotic effects [31,55]. In cells exposed to excess lipids, the removal of FFAs through increased TAG accumulation and β-oxidation appears to be a general cellular response to the lipotoxic effects of FA overload [31]. Thus, besides promoting TAG synthesis, OA also prevented palmitate-induced apoptosis in skeletal muscle cells by stimulating β-oxidation through elevation of the expression of CPT1, activation of AMPK and repression of the activity of ACC [72]. Similarly, hGX significantly increased the levels of two important β-oxidation enzymes, CPT1A and VLCAD, in MDA-MB-231 cells (Figure 7), in parallel with the high rate of LD formation, activation of AMPK (Figures 8A and 8B) and suppression of the induction of lipogenic enzymes, including ACC1 (Figure 7). Nevertheless, it is highly likely that, besides OA, other products of hGX phospholipid hydrolysis contribute to its effects in breast cancer cells, either by feeding metabolic pathways or by triggering cell signaling to various degrees [73]. Our results indicate that cPLA_2_α activation and LPA signaling (Additional file 5: Figure S4) are not important for the effects of hGX on MDA-MB-231 cells. However, the ability of rapamycin and indomethacin to partially suppress hGX-induced LD formation points to a possible role for AA in supporting LD formation through mTOR activation [49] and COX-dependent prostaglandin synthesis, respectively. Nevertheless, the contribution of AA-mediated signaling mechanisms to the changes in lipid metabolism induced by hGX sPLA_2_ in MDA-MB-231 cells is clearly minimal. Altogether, the results presented in this study suggest that FFAs, in particular OA, liberated from membrane phospholipids by the enzymatic activity of hGX sPLA_2_ are responsible for the observed alterations in lipid metabolism and the pro-survival effects induced by hGX in MDA-MB-231 breast cancer cells.

LDs, the intracellular neutral lipid storehouses until recently regarded as inert energy depots, are now regarded as complex organelles not only involved in the metabolic regulation of lipolysis and lipogenesis, but also in cell survival, apoptosis and cancer [3,5,31]. hGX sPLA_2_ induced robust TAG synthesis and LD formation in proliferating MDA-MB-231 cells (Figures 2C and 2E), but the effects on cell proliferation were modest (Additional file 1: Figure S1A). On the other hand, although LD formation was less pronounced in serum-deprived cells, the increase in cell proliferation (Figure 1A) and, in particular, the reduction in apoptosis (Figure 1B) were more significant. This suggests a mechanism by which the formed LDs provide energy, building blocks or signaling molecules to sustain cell survival during energy stress [3,5,31]. Consistent with this, although the LDs accumulated in hGX-treated proliferating cells exhibited a minimal immediate proliferative effect (Figures 1A and Additional file 1: Figure S1A), they conferred to the cells a marked survival advantage during long-term starvation in the absence of the sPLA_2_ (Figure 3B). The hGX-induced LD accumulation was accompanied by increased levels of perilipin 2 mRNA, while a decrease in its transcriptional level was observed 24 h after the cells were switched to serum-free medium (Figure 7A). This is in line with its suggested role in promoting TAG accumulation and blocking lipolysis [3,59], as well as with the reported correlation between TAG amount and perilipin 2 expression [57]. Since the transcription of β-oxidation genes was elevated almost in parallel with that of perilipin 2, it is conceivable that the FFAs released by hGX from membrane phospholipids are immediately partitioned between β-oxidation and TAG synthesis, which may contribute to cell survival by minimizing FFA toxicity [55]. However, since hGX-induced LDs were sufficient to prevent cell death in the absence of the sPLA_2_ (Figure 3B), the FFAs released following LD lipolysis are probably also involved in the hGX-induced changes in cell metabolism and survival [3,5]. Indeed, a cycle of FFA esterification and TAG lipolysis was required for FA-induced PPAR-mediated signaling responsible for mitochondrial gene expression and oxidative phosphorylation in cardiomyocytes [74]. Furthermore, PPAR activation by lipolytic FFAs modulated mitochondrial gene expression in brown adipose tissue, matching FA oxidation with supply [75]. In line with this, the hGX-induced alterations in gene expression were augmented when proliferating cells were switched to serum-free and sPLA_2_-free medium (Figure 7A), suggesting that they form the basis for the metabolic adaptations that enable the positive effects of hGX on cell survival. Under these conditions, the pro-survival effects of the pre-formed LDs were abolished if high concentrations of etomoxir were used to block β-oxidation and LD breakdown (Figures 6D and 6E), suggesting that TAG lipolysis followed by β-oxidation is critical for the pro-survival effects of hGX-induced LDs in MDA-MB-231 cells.

There is increasing evidence that CPT1 activity and β-oxidation contribute to the metabolic adaptations that enable cancer cell growth and survival [7]. Accelerated β-oxidation protects cancer cells from cell death induced by starvation or matrix detachment [6,8,10,35] by contributing ATP and generating NADPH to counteract the accumulation of ROS during metabolic stress [7-9,35]. Furthermore, the ability of etomoxir to block the positive effect of hGX on cancer cell survival (Figure 6) is in line with recent studies showing that etomoxir-mediated inhibition of β-oxidation leads to a reduction in cancer cell proliferation and increased sensitivity to cell death [9,35,53]. Additionally, apoptosis-induced mitochondrial damage leads to LD formation due to inhibition of β-oxidation and increased *de novo* lipid synthesis [76]. The opposite alterations in FA oxidation and synthesis induced by hGX sPLA_2_ in MDA-MB-231 cells may thus counteract the apoptosis-related changes and avert cell death. Therefore, the increased levels of CPT1A and VLCAD in hGX-treated cells, together with the ability of etomoxir to abrogate hGX-induced cell survival and induce cell death in starved MDA-MB-231 cells, strongly suggest that β-oxidation, and in particular CPT1 activity, is necessary for the positive effects of hGX on MDA-MB-231 cell proliferation and survival following serum withdrawal.

The central metabolic regulator AMPK responds to energy stress by suppressing ATP-consuming processes, including FA, cholesterol and TAG synthesis [61,63], while stimulating ATP-producing processes, such as glycolysis, mitochondrial biogenesis and β-oxidation [3,8,61]. The acute effects of AMPK activation in most cell types include a direct inactivation of ACC, leading to suppression of FA synthesis, and also to a reciprocal stimulation of CPT1 activity and β-oxidation due to reduction in malonyl-CoA levels [61]. Reduced expression and activity of AMPK have been found in many cancers, including primary breast tumors [77]. A metabolic tumor suppressor role has been demonstrated recently for AMPK in lymphoma, where it negatively regulates the Warburg effect and limits cancer cell growth [78]. However, AMPK can also support cancer cell survival and invasiveness [8,60], suggesting that its role in cancer is dependent on the cancer cell type and the pathophysiological context [8,79]. In this study, we show that the activity of hGX sPLA_2_ in invasive breast cancer cells leads to the activation of AMPK, suggesting that the kinase supports the pro-tumorigenic metabolic alterations induced by hGX sPLA_2_. Elevated phosphorylation of AMPK was detected in hGX-treated cells after 48 h of cell proliferation (Figures 8A and 8B) when neutral lipid accumulation reached maximal levels (Figure 2C) and the gene expression changes were significant (Figure 7A). Furthermore, etomoxir and triacsin C, which both attenuated hGX-induced LD formation, also prevented hGX-induced AMPK activation (Figures 8A and 8B). This suggests that the energy stress caused by rapid cell growth and proliferation combined with extensive FA activation, TAG synthesis and LD biogenesis in hGX-treated MDA-MB-231 cells leads to AMPK activation [29]. Accordingly, by mimicking cellular low energy status and inducing a several-fold higher increase in the level of phosphorylated AMPK relative to hGX (Figures 8A and 8B), the AMPK activator AICAR completely prevented hGX-induced LD formation (Figure 8C). This is consistent with the previously reported strong cytostatic effect of AICAR on MDA-MB-231 cells caused by suppression of DNA, protein and lipid synthesis [64]. It is thus possible that one of the important roles of AMPK in hGX-treated cells is to restore the energy balance by preventing further LD formation, by suppressing TAG synthesis, by phosphorylating glycerol-3-phosphate acyltransferase (GPAT) [63], and by stimulating lipolysis, presumably by activating adipose triglyceride lipase (ATGL/PNPLA2) [3,62], as well as β-oxidation. Apart from these immediate effects on lipid metabolism, the observed long-lasting transcriptional adaptations induced by hGX in MDA-MB-231 cells (Figure 7A) could also be mediated by AMPK. Namely, AMPK blocks SREBP-1 activity by direct phosphorylation [80] or through inhibition of mTOR [50], thus suppressing the transcription of its target genes, including *ACACA*, *FASN* and *SCD*[4], but also lowering SREBP-1 expression by reducing its auto-loop regulation [80]. Additionally, hGX-released polyunsaturated FAs may directly suppress the expression of SREBP-1 and its target genes [34], including *FASN* and *SCD*, whose inhibition has been shown to induce AMPK activation [4]. Also, elevated AMPK activity may induce the expression and activity of peroxisome proliferator-activated receptor-γ co-activator 1α (PGC1α) [7,61] to stimulate mitochondrial biogenesis and the transcription of β-oxidation genes, such as those encoding CPT1A and VLCAD. Similarly to the effects of hGX in MDA-MB-231 cells, increased rates of β-oxidation associated with AMPK phosphorylation, elevation of CPT1A mRNA and a decrease in lipogenesis due to inactivation of ACC have recently been implicated in the adipocyte-induced survival and metastasis of ovarian cancer cells [60]. Importantly, it has been shown that activation of AMPK in cancer cells during energy stress enables cell survival by blocking lipid synthesis through inactivation of ACC and elevating β-oxidation-dependent NADPH production to restore the redox balance [8]. Our results indicate that AMPK activation also supports survival of MDA-MB-231 cells, since AICAR displayed a strong anti-apoptotic effect in these cells (Figure 8E). Thus, the activation of AMPK by hGX in proliferating cells implicates AMPK in the coordination of the adaptation of MDA-MB-231 cell metabolism to the FAs derived from hGX membrane hydrolysis. Its association with the hGX sPLA_2_-induced LD formation and cell survival, however, remains to be confirmed.

Our results with etomoxir and bezafibrate, modulators of β-oxidation, suggest that β-oxidation supports the process of hGX-induced LD biogenesis in MDA-MB-231 cells, regardless of their metabolic and proliferative status (Figures 6A and 6C). It is, however, not clear how β-oxidation can support LD formation. Presumably, elevated β-oxidation may provide ATP and NADPH [7] for the energetically expensive process of LD formation [29], which, besides TAG synthesis, also requires alterations in FA, cholesterol and phospholipid synthesis and remodeling [30]. Although the simultaneous activity of FA synthesis and oxidation is controversial [7], a high β-oxidation flux could contribute to the cytosolic pool of acetyl-CoA molecules for *de novo* FA synthesis. Thus, despite the increased level of FFAs released by the sPLA_2_ from phospholipids and from TAGs through lipolysis, a low level of FA synthesis is probably still necessary for maintaining the proper FA composition of cell membranes and the membranes of LDs, in particular in proliferating cells [1,30]. Additionally, hGX may stimulate a cycle of FA esterification and lipolysis, as suggested for OA in MDA-MB-231 cells [29,42]. Since FA/TAG cycling requires high ACS activity, at the expense of ATP, to provide a continuous supply of FA-CoA, it may also contribute to the observed hGX-induced activation of AMPK [29]. In line with this, besides etomoxir, the ACS inhibitor triacsin C also partially blocked hGX-induced LD formation (Additional file 5: Figure S4) and AMPK activation (Figures 8A and 8B) in proliferating cells. We may thus speculate that, by supplying FFAs, hGX stimulates β-oxidation that in turn supports the anabolic branch of FA/TAG cycling, resulting in net LD accumulation and thus filling the LD energy reserves that can be used to support cell survival. Interestingly, recent studies revealing that mitochondria form contact sites with nascent LDs and participate in phospholipid and TAG synthesis during their biogenesis [30] are in line with a possible association between β-oxidation and LD formation. It is thus likely that hGX sPLA_2_ modulates the balance between the catabolic branch of glycerolipid metabolism, including TAG lipolysis and β-oxidation, and the anabolic processes, such as *de novo* FA synthesis, phospholipid remodeling and TAG synthesis. sPLA_2_ phospholipid hydrolysis, which may feed a variety of lipids into both branches, would thus induce metabolic alterations that lead to net LD accumulation and enable the pro-survival activity of hGX in MDA-MB-231 cells during prolonged serum deprivation.

The metabolic transformations induced by hGX sPLA_2_ in the highly invasive breast cancer cells, that include increased accumulation of cytosolic LDs, up-regulated β-oxidation and suppressed lipogenesis, resemble the effects of omental fat pad adipocytes that provide lipids for ovarian cancer cells to enable their growth and survival at the metastatic site [60]. Interestingly, when MDA-MB-231 and T-47D cells were exposed to the same primary human fat pad adipocytes they also accumulated large amounts of LDs and displayed increased invasive properties [60]. This suggests that the effects of hGX sPLA_2_ identified in this study could be pathophysiologically relevant. hGX sPLA_2_ may be secreted not only from breast cancer cells, but also from different cells in the tumor microenvironment, including inflammatory cells [12] and adipocytes [34], at primary tumor sites or at lipid-rich metastatic sites. It may then act in an autocrine or paracrine manner on cellular and extracellular phospholipids [12] to alter the availability of FFAs and induce metabolic transformations in cancer cells to support their survival, growth and metastatic potential. Furthermore, alterations in lipid metabolism and lipid accumulation within LDs in non-adipose tissue have been recognized as a major risk factor for the development of cancer and also other chronic diseases, such as metabolic syndrome, cardiovascular disease and diabetes [4,31]. Thus, the present study raises the possibility that modulation of cellular lipid metabolism by hGX and other sPLA_2_s may also contribute to some of these debilitating diseases.

## Conclusions

Several sPLA_2_s have been previously shown to affect the fate of cancer and other cells, however, their mechanisms of action at the cellular level are still unclear, have not been causally linked to eicosanoid or other lipid signaling, and have never been related to lipid droplets or alterations in basic lipid metabolism. We show in this study that hGX sPLA_2_, through the products of its enzymatic activity, induces LD formation and alters lipid metabolism in triple negative breast cancer cells, stimulating their proliferation and prolonging cell survival during growth factor deprivation. We provide evidence that the pro-tumorigenic effects of hGX are associated with activation of AMPK, suppression of lipogenesis and activation of β-oxidation, which is critical for the survival of hGX-treated MDA-MB-231 cells, most probably by contributing to the restoration of the energy and redox balance [1,7]. The results also suggest the intriguing possibility that hGX-induced elevated β-oxidation also supports the anabolic branch of FA/TAG cycling, leading to a net LD accumulation that in turn enables prolonged cell survival. Finally, the ability of hGX sPLA_2_ to act as a modulator of basic lipid metabolism and cancer cell survival is established. This could have important implications in elucidating the role of hGX and other sPLA_2_s, such as hGV and hGIII, in cancer and human pathophysiology in general.

## Materials and methods

### Materials

Cell cultures (MDA-MB-231, T-47D, MCF7, SK-BR-3, MCF-10A) and culture media (RPMI-1640, MEM, McCoy’s 5A) were from ATCC (USA). Mammary epithelial cell growth medium (MEGM) was from Lonza (USA) and fetal bovine serum (FBS), Dulbecco’s phosphate-buffered saline (DPBS), TrypLe Select and Opti-MEM from Life Technologies (USA). Varespladib (LY315920) was from Selleck Chemicals (USA) and fatty acid-free (FAF) BSA (#A7511), Nile red, tetramethylrhodamine, methyl ester (TMRM), etomoxir sodium salt hydrate, bezafibrate and S32826 from Sigma-Aldrich (USA). YO-PRO-1 iodide was from Life Technologies (USA); oleic acid and rapamycin were from Merck (Germany), 5-aminoimidazole-4-carboxamide ribonucleoside (AICAR) and pyrrolidine-2 (also called pyrrophenone) were from Cayman Chemical (USA), BrP-LPA was from Tebu-Bio (France), indomethacin and triacsin C were from Enzo Life Sciences (Switzerland). The phospho-AMPKα (Thr172) mAb (#2535), AMPKα mAb (#2603), acetyl-CoA carboxylase mAb (#3676), phospho-Akt (Ser473) mAb (#4060), Akt (pan) mAb (#4691) were from Cell Signaling Technology (USA). The SREBP-1 (#sc-367) and VLCAD antibodies (#sc-271225) were from Santa Cruz Biotechnology (USA), β-actin antibody (NB600-532) was from Novus Biologicals (UK). AZ-1 was provided by Prof. Michael H. Gelb (University of Washington, Seattle, USA) and corresponds to compound 22 in Connolly *et al.*[48]. The recombinant wild-type mammalian group IIA, V and X sPLA_2_s, the catalytically inactive mutant (H48Q) of mouse group X sPLA_2_ and the V31W mutant of the snake venom sPLA_2_ AtxA were prepared as described [13,46,81]. All other chemicals were of at least analytical grade and purchased from Sigma-Aldrich (USA) and Serva (Germany).

### Cell lines and culture conditions

The MDA-MB-231 and T-47D cell lines were cultured in RPMI-1640 medium supplemented with 10% FBS, and with 0.2 Units/ml of bovine insulin (Sigma-Aldrich, USA) in the case of the T-47D cell line. MCF7 cells were cultured in MEM with 10% FBS and 0.01 mg/ml bovine insulin, SK-BR-3 cells in McCoy's 5A medium supplemented with 10% FBS and the MCF-10A cell line in MEGM in the presence of 100 ng/ml cholera toxin (Sigma-Aldrich, USA) and without the supplement GA-1000 (Lonza, USA). In experiments using serum-deprived cells, FBS was replaced by 0.02–0.5% FAF BSA. Pharmacological agents were added to cell culture media at an appropriate concentration 1 h prior to the addition of recombinant sPLA_2_ and were present in the media for the duration of the treatment. The sPLA_2_ inhibitor varespladib was incubated with the enzyme in the appropriate medium at a concentration of 50 μM for 15 min and the mixture then added to cells. In experiments longer than 48 h, culture media was replenished by adding an aliquot from the stock inhibitor solution. Oleic acid was complexed to 0.5% FAF BSA or 10% FBS in culture medium before addition to cell culture.

### Real-time quantitative PCR (qPCR)

Cells were seeded in 6-well plates at a concentration of 1.5 × 10^5^ cells/well. 24 h later they were treated with 1 nM hGX in complete culture medium and incubated for an additional 48 h. The cells were washed with DPBS and incubated for an additional 48 h in serum-free medium containing 0.02% FAF BSA and harvested at desired time-points. Total RNA was extracted from cell lysates using TRIzol reagent (Life Technologies, USA) according to the manufacturer’s instructions and quantified using a NanoDrop Spectrophotometer (Thermo Scientific, Rockford, USA). RNA quality was assessed using an Agilent 2100 Bioanalyzer (USA). First strand cDNA was synthesized from 1 *μ*g of RNA using the High Capacity cDNA Reverse Transcription Kit with RNase Inhibitor (Life Technologies, USA) and random primers, according to the manufacturer’s instructions. qPCR reactions were carried out for all genes of interest and two reference genes (Additional file 2: Table S1) in each sample using LightCycler 480 SYBR Green I Master (Roche Applied Science, Germany) chemistry on a LightCycler 480 instrument (Roche Applied Science, Germany). All reactions were performed in a total volume of 5 *μ*l and contained 10 ng RNA equivalent cDNA and 250 nM of each set of primers. Thermal cycles were set at 95°C for 10 min, followed by 45 cycles of 95°C for 10 s, 60°C for 15 s and 72°C for 20 s. No template control reactions were included in the assays. PCR efficiencies were at least 80% for all primer pairs and a single melting peak was observed for each primer pair. Relative gene expression was calculated upon normalization to two reference genes and corrected for primer-specific PCR efficiency as described previously [82].

### Transient transfection

The full-length cDNA coding for hGX sPLA_2_[83] was cloned into the pcDNA3.1/D-V5-His-TOPO expression vector (Life Technologies, USA) according to manufacturer’s instructions. The hGX H48Q mutant was generated using the QuikChange II Site-Directed Mutagenesis Kit (Agilent Technologies, USA) following manufacturer’s instructions. For transient transfection, MDA-MB-231 cells were seeded in 24-well plates at a concentration of 1.5 × 10^5^ cells/well and incubated for 24 h in complete culture medium. Cells were transfected with 0.8 μg/well of plasmid DNA using 1.6 μl/well Lipofectamine 2000 (Life Technologies, USA) according to manufacturer’s instructions. Cell proliferation was measured 48 h after transfection. For determination of cell survival after serum-deprivation, cells were washed twice with serum-free medium containing 0.05% FAF BSA 24 h post-transfection, incubated in the same medium for an additional 96 h and analyzed using the TMRM/YO-PRO-1 cell death assay.

### Cell proliferation assay

Cells were plated in complete medium in 24-well culture plates at 6 × 10^4^ cells per well. After 24 h the medium was replaced with serum-free medium containing 0.1% BSA and the cells incubated for 48 h. Quiescent cells were then treated for 24 h with 10 nM hGX in serum-free medium with 0.1% BSA. The 5-ethynyl-2’-deoxyuridine (EdU) nucleoside analog was added at a final concentration of 10 μM for the last 6 h of cell treatment. Floating and attached cells were harvested together and stained with Click-iT EdU Alexa Fluor 488 Flow Cytometry Assay Kit (Life Technologies, USA) according to manufacturer's instructions. RNase A (Sigma-Aldrich, USA) was added to a final concentration of 200 μg/ml and cellular DNA was stained with 7-AAD (Life Technologies, USA) added to a final concentration of 10 μg/ml for 1 h. Samples were analyzed on a FACSCalibur flow cytometer equipped with a 488-nm Ar-ion laser using the CellQuest software (Becton Dickinson, USA). The logarithmic Alexa 488 fluorescence signal was collected using the FL-1 filter (530/30) and linear 7-AAD fluorescence signal was collected using the FL-3 filter (650LP). Samples were prepared in duplicate with analysis on 2 × 10^4^ events per sample.

### TMRM/YO-PRO-1 apoptosis assay

For survival assays, cells were seeded in 24-well culture plates at a concentration of 6 × 10^4^ cells/well (MDA-MB-231, T-47D), 3 × 10^4^ cells/well (MCF-10A) or 1 × 10^5^ cells/well (MCF7, SK-BR-3). After 24 h, cells were placed in their respective serum-free media with 0.02% FAF BSA for an additional 24 h, and treated with sPLA_2_ and effectors in serum-free medium with 0.02% FAF BSA for an additional 96 h (MDA-MB-231), 120 h (MCF7, MCF-10A, SK-BR-3) or 168 h (T-47D) and the cells harvested for analysis. To test the effect of pre-formed LDs on cell survival, MDA-MB-231 cells were plated in 24-well culture plates at a concentration of 3 × 10^4^ cells/well. Twenty-four hours later, the medium was discarded and 1 nM hGX in complete culture medium was added for an additional 48 h. hGX was removed by washing the cells twice with DPBS, the cells serum-starved in the presence of 0.02% FAF BSA for an additional 96 h and then harvested for analysis. The percentage of apoptotic cells was determined by TMRM/YO-PRO-1 staining using an adapted version of the protocol described previously [84]. Floating and adherent cells were combined, pelleted, resuspended in 100 μl of 150 nM TMRM solution in DPBS and incubated for 15 min in the dark at room temperature. YO-PRO-1 iodide was added to a final concentration of 50 nM for an additional 10 min. The cell suspension was diluted with 200 μl 0.1% BSA in DPBS and analyzed by flow cytometry. The YO-PRO-1 and TMRM fluorescence signals were collected using FL-1 (530/30) and FL-3 (650LP) filters, respectively. TMRM negative and YO-PRO-1 positive cells were considered apoptotic. Samples were prepared in duplicate and analyzed on 2 × 10^4^ events per sample.

### Nile red staining of lipid droplets

Cells were seeded in complete culture medium in 24-well plates at a concentration of 3 × 10^4^ cells/well (MDA-MB-231), 6 × 10^4^ cells/well (T-47D) or 10^5^ cells/well (MCF7). Twenty-four hours later, cells were treated with hGX in complete culture medium and incubated for at least 24 h prior to Nile red staining. For LD analysis of serum-deprived cells, the cells were treated as described above for the apoptosis assay. Cells were harvested, and the pellet resuspended in 500 μl of 1 μg/ml Nile red solution in DPBS and incubated in the dark for 10 min. Immediately after staining, the cells were analyzed by flow cytometry. The logarithmic fluorescence signal was collected using the FL-1 filter (530/30). Samples were prepared in duplicate and analyzed on 2 × 10^4^ events per sample.

### Triglyceride assay

MDA-MB-231 cells were seeded on 10-cm plates at a concentration of 1 × 10^6^ cells/well, grown for 24 h and treated with 1 nM hGX in complete culture medium for the next 48 h. The cells were harvested using a cell scraper, pelleted and cell lysates prepared for analysis of TAG content using the Triglyceride Fluorometric Assay Kit (Cayman Chemicals, USA) according to manufacturer’s instructions.

### Fluorescence microscopy

MDA-MB-231 cells were seeded on glass coverslips in 6-well plates at a density of 1.5 × 10^5^ cells/well, grown for 24 h and treated with 1 nM hGX in complete culture medium for the next 48 h. The cells were washed with DPBS and fixed with 4% paraformaldehyde in DPBS for 30 min, washed again, and stained with 1 μg/ml Nile red solution in DPBS for 10 min. After an additional washing step with DPBS, they were mounted on microscope slides using ProLong Gold Antifade Reagent with DAPI (Life Technologies, USA). The images were acquired using a Zeiss Axio Observer Z1 inverted microscope with a plan apochromatic objective (40×, 0.95 NA; Carl Zeiss, Germany), using 470/40 excitation and 525/50 emission filters for the Nile red signal and G365 excitation and 445/50 emission filters for the DAPI signal.

### Immunoblot analysis

Cell lysates were prepared by scraping adherent cells into 2× reducing protein-loading buffer (0.125 M Tris-HCl, pH 6.8; 4% SDS, 0.02% bromophenol blue, 20% glycerol, 100 mM DTT; Protein Loading Buffer Pack, Thermo Scientific, USA) with the addition of Halt Phosphatase Inhibitor Cocktail (Thermo Scientific, USA) and EDTA-Free Halt Protease Inhibitor Cocktail (Thermo Scientific, USA). The proteins were denatured by heating at 95°C for 10 min and total protein content determined using the Pierce 660 nm Protein Assay in the presence of 50 mM Ionic Detergent Compatible Reagent (Thermo Scientific, USA), using BSA as standard. Aliquots of 5–15 *μ*g of total protein were separated on a 7.5% or 10% SDS-PAGE gel and proteins transferred to a polyvinylidene difluoride (PVDF) membrane (Millipore, USA). After a 1 h-blocking step in 5% BSA in TBS or in 1% Western Blocking Reagent in TBS (Roche Applied Science, Germany), the membrane was incubated overnight at 4°C with gentle shaking in a solution of appropriately diluted primary antibody in 2.5% or 5% BSA in TBS/0.1% Tween-20 (TBST) or in 0.5% Western Blocking Reagent in TBS. After three washing steps in TBST, membranes were incubated with horseradish peroxidase (HRP)-conjugated secondary antibodies (Cell Signaling Technology or Jackson ImmunoResearch Laboratories, USA). After three additional washing steps, the proteins bands were visualized using Lumi-Light Western Blotting Substrate (Roche Applied Science, Germany) and Amersham Hyperfilm ECL films (GE Healthcare, UK). Band density was quantified by densitometric analysis using ImageJ software (National Institute of Health, USA).

### Statistical analysis

Data are presented as means ± SD. Prism software (GraphPad Software, USA) was used for statistical analysis, using Student’s *t*-test and one-way ANOVA with Bonferroni adjustment for multiple comparisons. P values lower than 0.05 were considered statistically significant.

## Abbreviations

AA: Arachidonic acid; ACC: Acetyl-CoA carboxylase; ACS: Acyl-CoA synthetase; ACSL3: Long-chain acyl-CoA synthetase 3; AICAR: 5-aminoimidazole-4-carboxamide ribonucleoside; AMPK: AMP-activated protein kinase; ATX: Autotaxin; AtxA: Ammodytoxin A; COX: Cyclooxygenase; cPLA2α: Cytosolic group IVA PLA_2_; CPT1: Carnitine *O*-palmitoyltransferase 1; CPT1A: The liver isoform of CPT1; EdU: 5-ethynyl-2’-deoxyuridine; FA: Fatty acid; FAF: Fatty acid-free; FAS: Fatty acid synthase; LD: Lipid droplet; LPA: Lysophosphatidic acid; LPC: Lysophosphatidylcholine; OA: Oleic acid; PC: Phosphatidylcholine; PI3K: Phosphatidylinositol 3-kinase; PLA2: Phospholipase A_2_; PPAR: Peroxisome proliferator-activated receptor; SCD-1: Stearoyl-CoA desaturase 1; sPLA2: Secreted phospholipase A_2_; SREBP-1: Sterol regulatory element-binding protein-1; TAG: Triacylglycerol; TMRM: Tetramethylrhodamine, methyl ester; VLCAD: Very long-chain acyl-CoA dehydrogenase. sPLA_2_ enzymes are abbreviated with a lowercase letter indicating the species of origin (h, human; m mouse) and with uppercase letters denoting the sPLA_2_ group (GIIA, GV, GX).

## Competing interests

The authors declare that they have no competing interests.

## Authors’ contributions

AP performed most of the experiments, VB performed qPCR analyses, cell survival and LD experiments with sPLA_2_s other than hGX, and the oleic acid release analyses. CP, TP and GL prepared recombinant sPLA_2_s. AP, VB and CP performed enzymatic assays on *E. coli* membranes. TP conceived the study and coordinated its design. TP and AP interpreted the results and wrote the manuscript. JP and GL participated in the coordination of the study. AP, VB, JP and GL participated in interpretation of the results and finalized the manuscript. All authors read and approved the final manuscript.

## Supplementary Material

Additional file 1: Figure S1 hGX and mGX stimulate the proliferation of breast cancer cells in an enzymatic activity-dependent manner. (A) MDA-MB-231 cells were cultured in complete medium in the presence of indicated concentrations of hGX for 72 h. (B) After serum deprivation for 48 h, MDA-MB-231 cells were treated with recombinant mGX, or its enzymatically-impaired mutant H48Q, at the indicated concentrations in serum-free medium containing 0.1% BSA for 24 h. Cell proliferation (A, B) was determined using the EdU incorporation assay on fixed cells with additional 7-AAD staining. The nucleoside analog EdU was added to a final concentration of 10 μM for the last 4 h (A) or 6 h (B) of treatment. (C) After serum deprivation for 24 h, MDA-MB-231 cells were treated with recombinant hGX (10 nM) in serum-free medium containing 0.02% FAF BSA for 96 h in the presence or absence of the pan-sPLA_2_ inhibitor varespladib (Var) at a final concentration of 50 μM. After 96 h, the adherent cells were washed and the number of viable cells was determined by trypan blue exclusion using a hemocytometer. Values are means ± SD of three experiments and results that are statistically significant over control samples are indicated (*, P < 0.05; one-way ANOVA with Bonferroni adjustment). Click here for file

Additional file 2: Table S1 Primers used in qPCR analysis. **Table S2.** Determination of hGX sPLA_2_ enzymatic activity in culture media of transfected MDA-MB-231 cells. MDA-MB-231 cells grown for 24 h in complete culture medium were transiently transfected with empty vector and plasmids encoding the wild-type hGX or catalytic-site mutant hGX(H48Q). The cells were then cultured in complete medium for an additional 72 h (FBS). Alternatively, the cells were washed 24 h post transfection and incubated in serum-free medium containing 0.05% FAF BSA for an additional 48 h (BSA). The concentration of hGX secreted in the culture medium at indicated time points was determined with the *in vitro* sPLA_2_ enzymatic assay using [^3^H]oleic acid-radiolabeled *E. coli* membranes as described in the Supplemental Method. Abbreviations: nd, not detected. Click here for file

Additional file 3: Figure S2 Treatment of proliferating MDA-MB-231 cells with hGX results in increased cell granularity. MDA-MB-231 cells were grown in complete medium for 24 h, then treated with hGX (100 nM) in complete medium for 48 h. The cells were harvested, resuspended in DPBS and their morphology analyzed by flow cytometry. Forward scatter (FSC) and side scatter (SSC) parameters were analyzed revealing a considerable increase in mean cell granularity (SSC) of hGX-treated cells (B) in comparison with control cells (A), indicating accumulation of cytoplasmic LDs. A representative scatter diagram is shown. Click here for file

Additional file 4: Figure S3 hGX sPLA_2_ releases oleic acid from MDA-MB-231 cells. MDA-MB-231 cells were labeled with [^3^H]OA and grown in complete medium in the presence of hGX (1 nM) for 24 h. [^3^H]OA release to the medium was determined as described in Supp. Methods. Values on the graph are means ± SD of three independent experiments performed in duplicate. Statistical significance is indicated (**, P = 0.0435; Student's *t*-test). Click here for file

Additional file 5: Figure S4 The effects of various pharmacological agents on hGX-induced changes in LD formation and survival of MDA-MB-231 cells. (A) MDA-MB-231 cells were grown in complete medium for 24 h, then treated with hGX (1 nM) in complete medium for 48 h in the presence or absence of indomethacin (Indo; 50 μM), pyrrolidine-2 (Pyr-2; 5 μM), AZ-1 (10 μM), rapamycin (Rapa; 1 μM), S32826 (10 μM), BrP-LPA (10 μM), triacsin C (TrC; 2 μM). Levels of LDs were determined by Nile red staining and normalized to control samples. LD content was significantly greater after hGX treatment, in spite of the presence of inhibitors. Indomethacin, rapamycin and triacsin C significantly attenuated the hGX-induced increase in LD content. Pyrrolidine-2 alone caused an increase in LD content over control values (#, P < 0.001; one-way ANOVA with Bonferroni adjustment) and, in combination with hGX, even potentiated its ability to induce LDs. (B) Quiescent MDA-MB-231 cells were treated with hGX (10 nM) in serum-free medium containing 0.02% FAF BSA for 96 h in the presence or absence of indomethacin (50 μM) and of pyrrolidine-2 (1 μM). The percentage of apoptotic cells was determined by the TMRM/YO-PRO-1 apoptosis assay. Indomethacin and pyrrolidine-2 did not attenuate the hGX-induced pro-survival effect, though pyrrolidine-2 exerted some mild toxicity in serum-starved cells. Values are means ± SD of at least two experiments and results that are statistically significant over control samples are indicated (*, P < 0.05; **, P < 0.01; ***, P < 0.001; one-way ANOVA with Bonferroni adjustment). Click here for file

Additional file 6: Figure S5 Non-toxic concentrations of etomoxir prevent the hGX-induced pro-survival effect. Serum-starved MDA-MB-231 cells were treated with hGX (10 nM) in serum-free medium containing 0.02% FAF BSA for 96 h in the presence or absence of different concentrations of etomoxir (Eto) as indicated. The percentage of apoptotic cells was determined by the TMRM/YO-PRO-1 apoptosis assay. The pro-survival effect of hGX was abolished in the presence of 20 μM and 50 μM concentrations of etomoxir, which were not toxic to control MDA-MB-231 cells. Values are means ± SD of three experiments performed in duplicate and results that are statistically significant over control samples are indicated (*, P < 0.05; ***, P < 0.001; one-way ANOVA with Bonferroni adjustment). Click here for file
