# Supplementary figures and images for: Group X secreted phospholipase A2 induces lipid droplet formation and prolongs breast cancer cell survival
Source: Mol Cancer. 2013 Sep 27;12:111. doi: 10.1186/1476-4598-12-111 (PMC3852912; doi:10.1186/1476-4598-12-111)

A

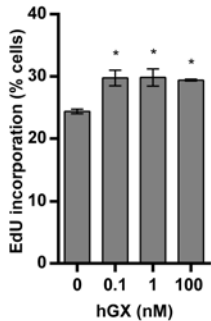

B

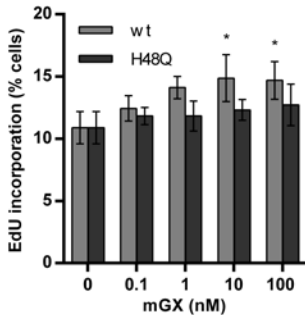

C

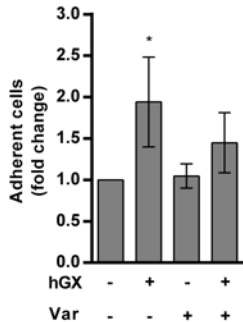

Fig. S1

Supplement: Additional file 1: Figure S1 — hGX and mGX stimulate the proliferation of breast cancer cells in an enzymatic activity-dependent manner. (A) MDA-MB-231 cells were cultured in complete medium in the presence of indicated concentrations of hGX for 72 h. (B) After serum deprivation for 48 h, MDA-MB-231 cells were treated with recombinant mGX, or its enzymatically-impaired mutant H48Q, at the indicated concentrations in serum-free medium containing 0.1% BSA for 24 h. Cell proliferation (A, B) was determined using the EdU incorporation assay on fixed cells with additional 7-AAD staining. The nucleoside analog EdU was added to a final concentration of 10 μM for the last 4 h (A) or 6 h (B) of treatment. (C) After serum deprivation for 24 h, MDA-MB-231 cells were treated with recombinant hGX (10 nM) in serum-free medium containing 0.02% FAF BSA for 96 h in the presence or absence of the pan-sPLA2 inhibitor varespladib (Var) at a final concentration of 50 μM. After 96 h, the adherent cells were washed and the number of viable cells was determined by trypan blue exclusion using a hemocytometer. Values are means ± SD of three experiments and results that are statistically significant over control samples are indicated (*, P < 0.05; one-way ANOVA with Bonferroni adjustment). [file 1476-4598-12-111-S1.pdf]

A

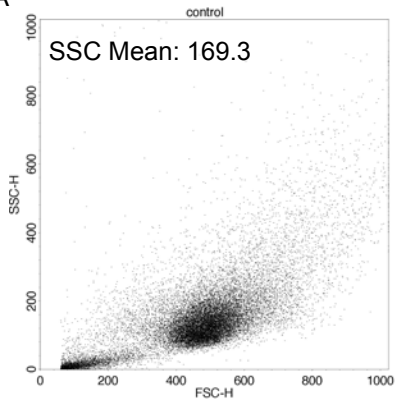

B

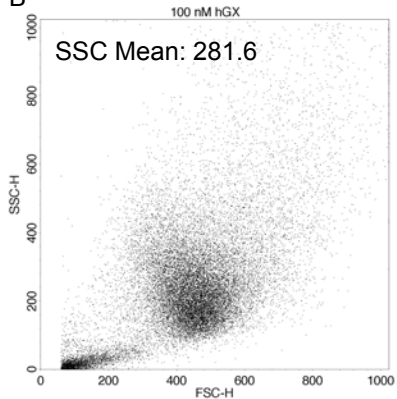

Fig. S2

Supplement: Additional file 3: Figure S2 — Treatment of proliferating MDA-MB-231 cells with hGX results in increased cell granularity. MDA-MB-231 cells were grown in complete medium for 24 h, then treated with hGX (100 nM) in complete medium for 48 h. The cells were harvested, resuspended in DPBS and their morphology analyzed by flow cytometry. Forward scatter (FSC) and side scatter (SSC) parameters were analyzed revealing a considerable increase in mean cell granularity (SSC) of hGX-treated cells (B) in comparison with control cells (A), indicating accumulation of cytoplasmic LDs. A representative scatter diagram is shown. [file 1476-4598-12-111-S3.pdf]

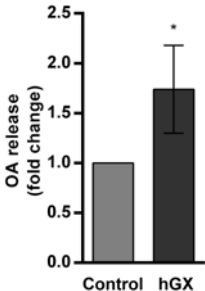

Fig. S3

Supplement: Additional file 4: Figure S3 — hGX sPLA2 releases oleic acid from MDA-MB-231 cells. MDA-MB-231 cells were labeled with [3H]OA and grown in complete medium in the presence of hGX (1 nM) for 24 h. [3H]OA release to the medium was determined as described in Supp. Methods. Values on the graph are means ± SD of three independent experiments performed in duplicate. Statistical significance is indicated (**, P = 0.0435; Student's t-test). [file 1476-4598-12-111-S4.pdf]

A

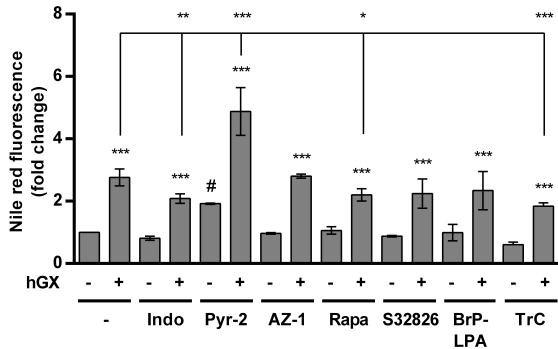

B

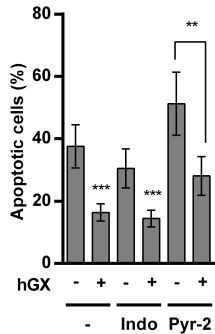

Fig. S4

Supplement: Additional file 5: Figure S4 — The effects of various pharmacological agents on hGX-induced changes in LD formation and survival of MDA-MB-231 cells. (A) MDA-MB-231 cells were grown in complete medium for 24 h, then treated with hGX (1 nM) in complete medium for 48 h in the presence or absence of indomethacin (Indo; 50 μM), pyrrolidine-2 (Pyr-2; 5 μM), AZ-1 (10 μM), rapamycin (Rapa; 1 μM), S32826 (10 μM), BrP-LPA (10 μM), triacsin C (TrC; 2 μM). Levels of LDs were determined by Nile red staining and normalized to control samples. LD content was significantly greater after hGX treatment, in spite of the presence of inhibitors. Indomethacin, rapamycin and triacsin C significantly attenuated the hGX-induced increase in LD content. Pyrrolidine-2 alone caused an increase in LD content over control values (#, P < 0.001; one-way ANOVA with Bonferroni adjustment) and, in combination with hGX, even potentiated its ability to induce LDs. (B) Quiescent MDA-MB-231 cells were treated with hGX (10 nM) in serum-free medium containing 0.02% FAF BSA for 96 h in the presence or absence of indomethacin (50 μM) and of pyrrolidine-2 (1 μM). The percentage of apoptotic cells was determined by the TMRM/YO-PRO-1 apoptosis assay. Indomethacin and pyrrolidine-2 did not attenuate the hGX-induced pro-survival effect, though pyrrolidine-2 exerted some mild toxicity in serum-starved cells. Values are means ± SD of at least two experiments and results that are statistically significant over control samples are indicated (*, P < 0.05; **, P < 0.01; ***, P < 0.001; one-way ANOVA with Bonferroni adjustment). [file 1476-4598-12-111-S5.pdf]

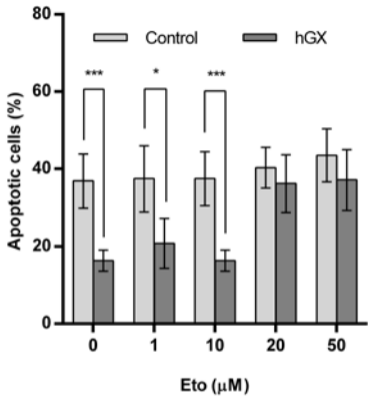

Fig. S5

Supplement: Additional file 6: Figure S5 — Non-toxic concentrations of etomoxir prevent the hGX-induced pro-survival effect. Serum-starved MDA-MB-231 cells were treated with hGX (10 nM) in serum-free medium containing 0.02% FAF BSA for 96 h in the presence or absence of different concentrations of etomoxir (Eto) as indicated. The percentage of apoptotic cells was determined by the TMRM/YO-PRO-1 apoptosis assay. The pro-survival effect of hGX was abolished in the presence of 20 μM and 50 μM concentrations of etomoxir, which were not toxic to control MDA-MB-231 cells. Values are means ± SD of three experiments performed in duplicate and results that are statistically significant over control samples are indicated (*, P < 0.05; ***, P < 0.001; one-way ANOVA with Bonferroni adjustment). [file 1476-4598-12-111-S6.pdf]
