# Supplementary material for: Group X secreted phospholipase A2 induces lipid droplet formation and prolongs breast cancer cell survival
Source: Mol Cancer. 2013 Sep 27;12:111. doi: 10.1186/1476-4598-12-111 (PMC3852912; doi:10.1186/1476-4598-12-111)
Supplement: Additional file 2: Table S1 — Primers used in qPCR analysis. Table S2. Determination of hGX sPLA2 enzymatic activity in culture media of transfected MDA-MB-231 cells. MDA-MB-231 cells grown for 24 h in complete culture medium were transiently transfected with empty vector and plasmids encoding the wild-type hGX or catalytic-site mutant hGX(H48Q). The cells were then cultured in complete medium for an additional 72 h (FBS). Alternatively, the cells were washed 24 h post transfection and incubated in serum-free medium containing 0.05% FAF BSA for an additional 48 h (BSA). The concentration of hGX secreted in the culture medium at indicated time points was determined with the in vitro sPLA2 enzymatic assay using [3H]oleic acid-radiolabeled E. coli membranes as described in the Supplemental Method. Abbreviations: nd, not detected. [file 1476-4598-12-111-S2.doc]

**ADDITIONAL FILE 2**

**Group X secreted phospholipase A_2_ induces lipid droplet formation and prolongs breast cancer cell survival**

**Supplementary Methods**

***In vitro* assay of sPLA_2_ enzymatic activity using [^3^H]oleic acid-radiolabeled *E. coli* membranes**

sPLA_2_ enzymatic activity was assayed at 37 °C for 1 h in 300 µl of assay buffer (0.1 M Tris-HCl, pH 8.0; 10 mM CaCl_2_, 0.1% BSA) with the addition of ~100,000 dpm of radiolabeled *E. coli* membranes [1]. Sample volume was adjusted in proportion to the amount of added sPLA_2_ and ranged from 10–50 µl. After the appropriate incubation time, reactions were stopped by the addition of 300 µl of stop buffer (0.1 M EDTA, 0.2% FAF BSA). Samples were then centrifuged (3 min; 14,000 rpm) and the supernatant was submitted to scintillation counting.

**sPLA_2_-induced oleic acid release from adherent cells**

MDA-MB-231 cells were plated in complete medium in 24-well culture plates at 6 × 10^4^ cells/well. Twenty four hours later, the cells were labeled by replenishing the medium with fresh complete medium containing 0.01 µCi [^3^H]OA per well. After 24 h the cells were washed three times with fresh complete medium and incubated for another 24 h in complete medium containing 1 nM hGX. Supernatants were removed and centrifuged for 5 min at 14,000 rcf to pellet dislodged cells and the adherent cells collected. The percentage of total [^3^H]OA released to the medium was calculated as 100 × (dpm in medium)/(dpm in medium × cell-associated dpm), determined by scintillation counting. Samples were prepared in duplicate.

**Supplementary Table 1.** Primers used in qPCR analysis.

| **Gene symbol** | **Accession #** | **Forward primer** | **Reverse primer** | **Reference** |
| --- | --- | --- | --- | --- |
| **ACACA** | NM_198834 | GGATGGTGTTCACTCGGTAATAGA | GGGTGATATGTGCTGCGTCAT | [2] |
| **ACADVL** | NM_000018 | ACCCGTCCGTGCTCAACGAA | CCAAGTGGTCTCCTCCACCAT | [3] |
| **ACSL3** | NM_004457 | CCCCTGAAACTGGTCTGGTG | TCCGCCTGGTAATGTGTTTTAA | [4] |
| **CPT1A** | NM_001876 | CCTCCAGTTGGCTTATCGTG | TTCTTCGTCTGGCTGGACAT | [5] |
| **FASN** | NM_004104 | AACTCCAAGGACACAGTCACCAT | CAGCTGCTCCACGAACTCAA | [6] |
| **HMGCR** | NM_000859 | ACAATAAGATCTGTGGTTGGAATTATGA | GCTATGCATCGTGTTATTGTCAGAA | [7] |
| **PLIN2** | NM_001122 | AGTATCCCTACCTGAAGTCTGTG | CCCCTTACAGGCATAGGTATTG | [8] |
| **SCD** | NM_005063 | CCTAGAAGCTGAGAAACTGGTGA | ACATCATCAGCAAGCCAGGT | [9] |
| **SF3A1** | NM_005877 | Not available | Not available | PrimerDesign (UK) |
| **SREBF1** | NM_001005291 | GGATTGCACTTTCGAAGACATG | AGCATAGGGTGGGTCAAATAGG | [7] |
| **TOP1** | NM_003286 | CCCTGTACTTCATCGACAAGC | CCACAGTGTCCGCTGTTTC | [10] |

**Supplementary Table 2.** Determination of hGX sPLA_2_ enzymatic activity in culture media of transfected MDA-MB-231 cells. MDA-MB-231 cells grown for 24 h in complete culture medium were transiently transfected with empty vector and plasmids encoding the wild-type hGX or catalytic-site mutant hGX(H48Q). The cells were then cultured in complete medium for an additional 72 h (FBS). Alternatively, the cells were washed 24 h post transfection and incubated in serum-free medium containing 0.05% FAF BSA for an additional 48 h (BSA). The concentration of hGX secreted in the culture medium at indicated time points was determined with the *in vitro* sPLA_2_ enzymatic assay using [^3^H]oleic acid-radiolabeled *E. coli* membranes as described in the Supplemental Method. Abbreviations: nd, not detected.

|  | [sPLA_2_] (pM) | | | | | |
| --- | --- | --- | --- | --- | --- | --- |
|  | 24 h | | 48 h | | 72 h | |
| pDNA | FBS | BSA | FBS | BSA | FBS | BSA |
| Empty vector | nd | nd | nd | nd | nd | nd |
| hGX | 255 ± 15 | - | 504 ± 17 | 247 ± 84 | 522 ± 19 | 326 ± 119 |
| hGX(H48Q) | nd | - | nd | nd | nd | nd |

**Supplementary References**

1. Ancian P, Lambeau G, Mattéi MG, Lazdunski M: **The human 180-kDa receptor for secretory phospholipases A_2_. Molecular cloning, identification of a secreted soluble form, expression, and chromosomal localization**. *J Biol Chem* 1995, **270**:8963–8970.

2. Kursawe R, Eszlinger M, Narayan D, Liu T, Bazuine M, Cali AMG, D'Adamo E, Shaw M, Pierpont B, Shulman GI, Cushman SW, Sherman A, Caprio S: **Cellularity and adipogenic profile of the abdominal subcutaneous adipose tissue from obese adolescents: association with insulin resistance and hepatic steatosis.** *Diabetes* 2010, **59**:2288–2296.

3. Djouadi F, Aubey F, Schlemmer D, Ruiter JPN, Wanders RJA, Strauss AW, Bastin J: **Bezafibrate increases very-long-chain acyl-CoA dehydrogenase protein and mRNA expression in deficient fibroblasts and is a potential therapy for fatty acid oxidation disorders.** *Hum Mol Genet* 2005, **14**:2695–2703.

4. Sandoval A, Fraisl P, Arias-Barrau E, Dirusso CC, Singer D, Sealls W, Black PN: **Fatty acid transport and activation and the expression patterns of genes involved in fatty acid trafficking.** *Arch Biochem Biophys* 2008, **477**:363–371.

5. Kobayashi M-A, Watada H, Kawamori R, Maeda S: **Overexpression of acetyl-coenzyme A carboxylase beta increases proinflammatory cytokines in cultured human renal proximal tubular epithelial cells.** *Clin Exp Nephrol* 2010, **14**:315–324.

6. Qiao S, Pennanen P, Nazarova N, Lou Y-R, Tuohimaa P: **Inhibition of fatty acid synthase expression by 1alpha,25-dihydroxyvitamin D3 in prostate cancer cells.** *J Steroid Biochem Mol Biol* 2003, **85**:1–8.

7. Abildayeva K, Jansen PJ, Hirsch-Reinshagen V, Bloks VW, Bakker AHF, Ramaekers FCS, de Vente J, Groen AK, Wellington CL, Kuipers F, Mulder M: **24(S)-hydroxycholesterol participates in a liver X receptor-controlled pathway in astrocytes that regulates apolipoprotein E-mediated cholesterol efflux.** *J Biol Chem* 2006, **281**:12799–12808.

8. Yin P, Roqueiro D, Huang L, Owen JK, Xie A, Navarro A, Monsivais D, Coon JS, Kim JJ, Dai Y, Bulun SE: **Genome-wide progesterone receptor binding: cell type-specific and shared mechanisms in T47D breast cancer cells and primary leiomyoma cells.** *PLoS ONE* 2012, **7**:e29021.

9. Yee JK, Phillips SA, Allamehzadeh K, Herbst KL: **Subcutaneous adipose tissue fatty acid desaturation in adults with and without rare adipose disorders.** *Lipids Health Dis* 2012, **11**:19.

10. Mounier CM, Wendum D, Greenspan E, Fléjou J-F, Rosenberg DW, Lambeau G: **Distinct expression pattern of the full set of secreted phospholipases A_2_ in human colorectal adenocarcinomas: sPLA_2_-III as a biomarker candidate.** *Br J Cancer* 2008, **98**:587–595.
